# Data-Driven Predetermination of Cu Oxidation State in Copper Nanoparticles: Application to the Synthesis by Laser Ablation in Liquid

**DOI:** 10.1021/jacs.3c09158

**Published:** 2023-10-31

**Authors:** Runpeng Miao, Michael Bissoli, Andrea Basagni, Ester Marotta, Stefano Corni, Vincenzo Amendola

**Affiliations:** Department of Chemical Sciences, University of Padova, 35131 Padova, Italy

## Abstract

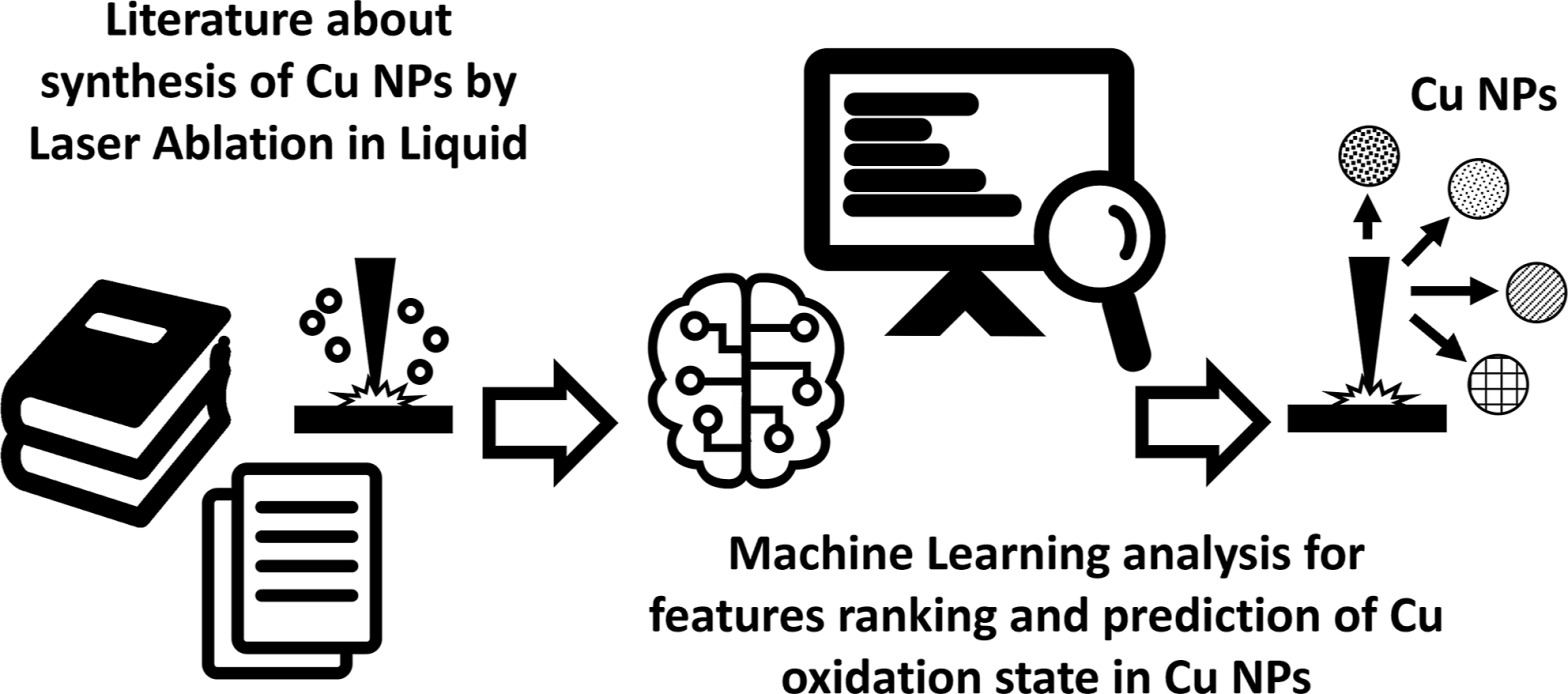

Copper-based nanocrystals are reference nanomaterials for integration into emerging green technologies, with laser ablation in liquid (LAL) being a remarkable technique for their synthesis. However, the achievement of a specific type of nanocrystal, among the whole library of nanomaterials available using LAL, has been until now an empirical endeavor based on changing synthesis parameters and characterizing the products. Here, we started from the bibliographic analysis of LAL synthesis of Cu-based nanocrystals to identify the relevant physical and chemical features for the predetermination of copper oxidation state. First, single features and their combinations were screened by linear regression analysis, also using a genetic algorithm, to find the best correlation with experimental output and identify the equation giving the best prediction of the LAL results. Then, machine learning (ML) models were exploited to unravel cross-correlations between features that are hidden in the linear regression analysis. Although the LAL-generated Cu nanocrystals may be present in a range of oxidation states, from metallic copper to cuprous oxide (Cu_2_O) and cupric oxide (CuO), in addition to the formation of other materials such as Cu_2_S and CuCN, ML was able to guide the experiments toward the maximization of the compounds in the greatest demand for integration in sustainable processes. This approach is of general applicability to other nanomaterials and can help understand the origin of the chemical pathways of nanocrystals generated by LAL, providing a rational guideline for the conscious predetermination of laser-synthesis parameters toward the desired compounds.

## Introduction

Nanomaterials play a major role in the global challenge toward the solution of the environmental crisis and the development of a circular economy, as indicated by the United Nations Sustainable Development Goals and the European Union Circular Economy Action Plan.^1,2^ Among the nanomaterials which are indispensable components for the most advanced technologies integrated with renewable energy sources,^2,3^ copper-based nanoparticles (NPs) have gained substantial attention.^4−6^ As other transition metals, Cu is earth-abundant, and its oxides are considered inert, nontoxic, stable, and low-cost.^4−6^ Copper oxides are intensely studied as heterogeneous catalysts for their good reactivity and selectivity in numerous oxidation and reduction reactions.^7,8^ Metallic copper NPs and copper oxide NPs doped with metallic clusters are the most selective electrocatalysts for the conversion of CO_2_ into chemical feedstock, possibly powered by renewable energy.^9−11^ CuO NPs are versatile p-type semiconductors because of the ease of manipulation of its band gap by quantum confinement, and CuO was one of the earliest proposed wide-bandgap oxides for all-oxide photovoltaic cells.^6,12^ Copper oxide NPs emerged as a prominent material in solar thermal conversion research.^13^ Nano-Cu_2_O is suitable for the absorbing layer of all-oxide photovoltaic cells,^14−16^ for cathodes in dye-sensitized solar cells,^17^ as self-healing photocathode in photoelectrochemical hydrogen evolution,^18^ or the hole-transport layers in perovskite solar cells,^19^ while copper sulfides are efficient photo- and electro-catalysts.^20^ The interest in Cu-based NPs goes beyond energy materials because the implementation in supercapacitors,^21^ gas sensors,^22^ or nanofluids is in an advanced stage,^23^ and Cu NPs are among the most studied antimicrobial agents in the fight against antibiotic-resistant bacteria, which constitute one of the main global challenges for humankind.^24,25^

It is thus of utmost importance to develop new sustainable and efficient synthetic technologies for the realization of the type of Cu nanocrystals exploited in the next-generation catalytic processes, photovoltaic devices, environment remediation protocols, and any other technology for a sustainable future. Laser ablation in liquid (LAL) is renowned for the production of a library of additive-free nanocrystals by a green, scalable, and economically feasible procedure,^26−28^ thus standing as an excellent candidate for the synthesis of Cu NPs. In LAL, a bulk target immersed in a liquid solution is continuously ablated with laser pulses to produce a colloidal array of nanomaterials. Compared to other physical and chemical synthetic protocols reported to date, LAL has the advantages of avoiding complex and time-consuming preparation steps, high temperatures or pressures, vacuum, expensive starting materials, and toxic solvents and solutes.^28,29^ Moreover, the NPs are obtained in the liquid phase without the need for additives or surfactants instead of forming an agglomerated powder or a solution of ligand-coated nanocrystals. This avoids cumbersome cleaning steps to remove undesired byproducts and chemical impurities or residuals, which significantly impact the optical, electronic, and catalytic properties.^6,15^

With LAL, Cu, Cu_2_O, CuO, Cu_2_S, and CuCN NPs have been synthesized using a metallic Cu target while varying the laser source, setup, solvent, and solutes.^20,30−35^ Unfortunately, the LAL of Cu targets usually produces a mixture of these different phases. Picking just a specific compound is a rather empirical endeavor based on changing the LAL synthesis parameters, which is particularly complex in the case of compounds with intermediate oxidation states such as Cu_2_O. In the case of Cu-based NPs, the identification of the appropriate LAL conditions for a specific compound needs accurate quantitative assessment of the products, best achieved by quantitative analysis of the X-ray diffraction (XRD) pattern, and may still lead to misleading results due to aging or manipulation of the products before structural analysis (e.g., heating and air exposure), which ineluctably alter the oxidation state and the morphology of the Cu nanocrystals.^32,33,35,36^ Another critical aspect is the purity and aging of the bulk metal target used for LAL, which may undergo oxidation with a drastic change in the synthesis conditions.^28,29^

Despite the fact that in the last decades, LAL has been applied to the production of a variety of nanocrystals and with a wide range of experimental parameters, a systematic analysis of the relation of these parameters with synthesis products using a big-data approach has never been attempted, even starting from the identification of trivial linear correlations. In fact, the mastering and the in-deep understanding of the LAL process toward advanced nanocrystal predetermination and synthesis are still far from being achieved, and the understanding of the relation between the products and the synthesis parameters has remained prevalently empirical to date.^37−40^ In chemical synthesis, one successful strategy for the accelerated identification of the most effective synthesis conditions relies on automation.^41,42^ Despite LAL being amenable to remote control and full automation,^43,44^ this is possible only after fixing the laser source parameters and a restricted list of solvents and solutes, making process optimization through automation practically unfeasible. The intrinsic incompatibility of this technique with an automatic multiparametric assessment has left the wide space of fundamental LAL parameters (laser source, setup, solvent, solutes) mostly unexplored until now. Molecular dynamics succeeded so far in describing the main dynamic steps of matter ejection from the target and early stage of NP formation, but it was still unable to catch the complex chemistry of the whole process.^39,45^ This chemistry has been drafted in seminal studies based on empirical observation^46−49^ and only recently became the subject of time-resolved investigations, which substantiated the hypothesis based on ex situ results with advanced in situ experiments.^37,50^

Nowadays, a data-driven approach starting from the known literature^42,51,52^ is an indispensable strategy to orient the synthetic efforts through the space of LAL parameters until finding a reliable track for the synthesis of a specific Cu compound without resorting to time- and resource-intensive experiments. The first examples of nanocrystals generated by LAL are dated back to the 90s,^28,29,53^ and a large database of synthetic conditions is available for the identification of the experimental parameters correlated with each product output.

Linear regression analysis is the easiest way to quantitatively demonstrate the existence of a correlation in a data set of (feature, output) couples, where the feature is a physical quantity connected to an experimental parameter, and the output is a physical quantity identifying the outcome of the experimental process.^52^ Indeed, linear regression is successful only when cross-correlations of experimental parameters are not crucial for the output.^29,46^ This is not the case for laser synthesis of colloids because the space of features designed by laser, setup, solvent, and solute requires a deeper analytical approach capable of tracking nontrivial correlations between features and output and tolerating anomalies in the database created from the literature.

Machine learning (ML) techniques are renowned for their ability to expand the dimensional scale of the multiparametric analysis and for their consequent enormous potential in the identification of hidden correlations in complex systems.^42,51,54,55^ There are a variety of ML models, which efficiently approximate arbitrarily complex nonlinear relationships between variables.^16,41,56^ Recently, starting from appropriate databases, ML significantly contributed to the development and understanding of nanomaterials, guiding the optimization of synthetic protocols and quick material characterization on the experimental side,^9,16,52,57−61^ and to the calculation of next-generation force fields with accuracies similar to ab initio calculations on the modeling side.^10,51,62,63^ All this lets the research on materials science rapidly enter the data-driven age.^42^ The backbone of all ML approaches is materials data, intended as both size and quality of the data set.^42,52,64−66^

Here, we report on the first example of the application of regression analysis and ML to LAL synthesis. We realized a database with bibliographic data about Cu-based nanocrystals synthesized by LAL. Linear regression analysis and ML were applied to this database to identify the relevant features leading to a specific Cu oxidation state and predict a suitable set of parameters for harvesting the desired product. In this way, the linear regression provided basic information to understand the origin of the chemical pathways of nanomaterials generated by LAL, leading to the identification of a LAL equation that depends on the relevant features for determining the oxidation state of Cu in NPs. Only the ML tools, however, unlocked the cross-correlations among dominant features and allowed the prediction of the product oxidation state, in fair agreement with novel experimental results not included in the original database. In particular, the ML analysis succeeded in guiding the experiments to the achievement of intermediate Cu oxidation states (Cu(I) compounds), which are the most difficult to obtain by LAL when compared with metal Cu and CuO NPs. Through this study, LAL becomes an even more versatile and powerful method for the generation of Cu-based nanocrystals of great interest for integration in sustainable processes ranging from electrocatalysis to photocatalysis, photovoltaic cells, and many others. Moreover, the approach is of general applicability to other nanomaterials obtained by LAL, and it is expected to pave the way to the conscious predetermination of laser-synthesis parameters toward the desired compounds.

## Results

### Database Building and Identification of Features

**Figure 1:**
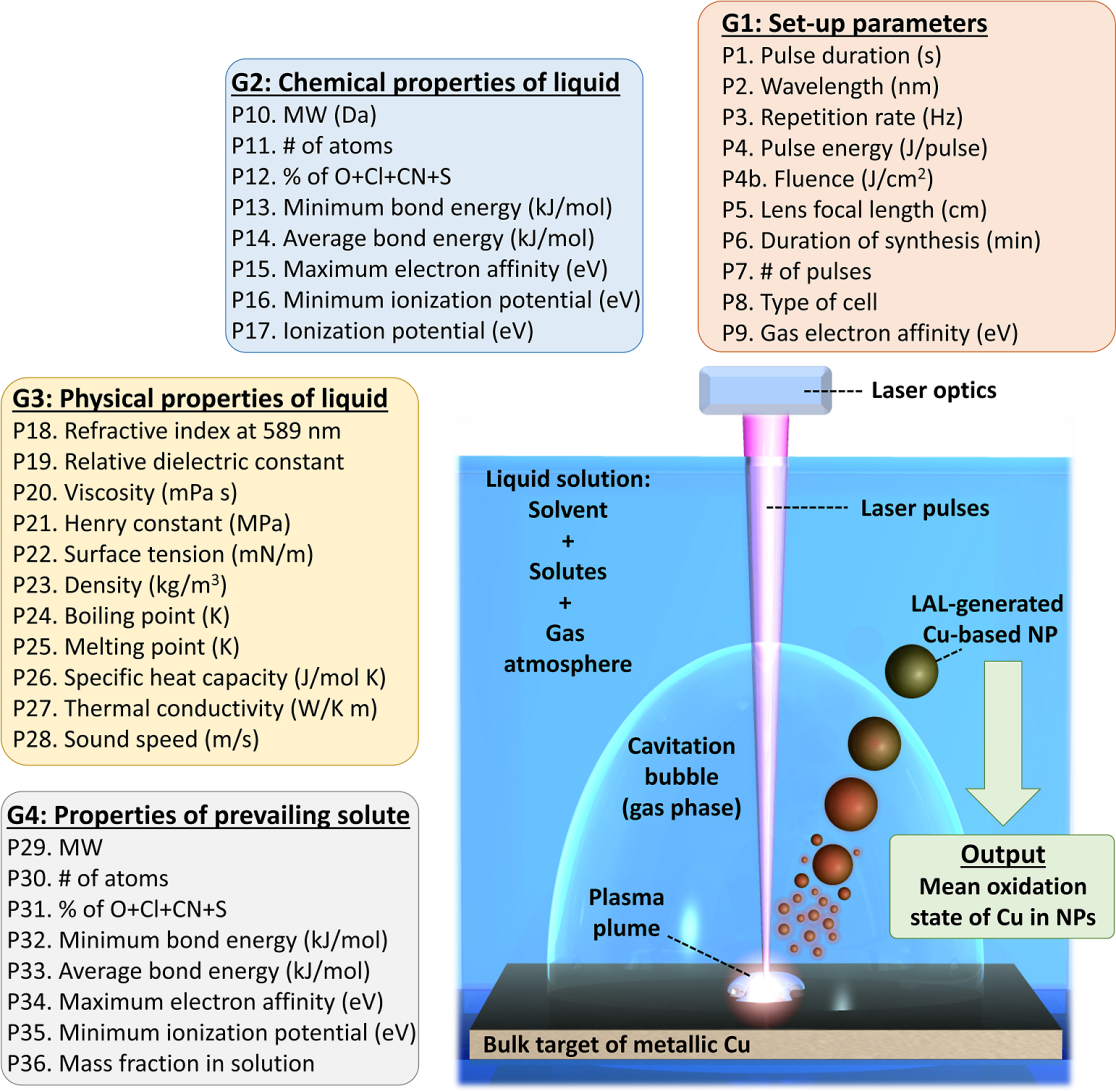
Sketch of the LAL process and list of the four groups G1–G4 of features derived from the experimental parameters adopted in the literature for the laser synthesis of Cu-based NPs from a metal Cu plate in liquid.

The synthesis by LAL takes place through a hierarchical series of chemical and physical consecutive processes, which are spatially anisotropic and partially overlapped in time on a submicrosecond time scale (Figure 1).^29,38,46^ In the majority of LAL experiments, they are as follows: (i) the absorption of the laser beam by the bulk target; (ii) the generation of two counterpropagating shockwaves toward the target and the above liquid;^38,67^ (iii) the ejection of the ablated material as a mixture of vapor and liquid drops forming a nonequilibrium plasma plume due to the explosive decomposition of target surface;^39,45,68−70^ (iv) the formation of a liquid vapor layer at the interface with the ablation plume, where mixing between the solution and target species begins;^38,39,45^ (v) the evolution of the vapor layer into a cavitation bubble, where the NPs are confined during their further growth and chemical transformation by reaction with the other species from the ablation plume and with liquid vapors;^29,37,38,46^ it is worth noting that, in addition to the target species, the thin liquid layer in contact with the ablation plume also undergoes to molecular fragmentation and ionization, becoming part of the ablation plume and of the interior of the cavitation bubble;^29,38,46^ (vi) the collapse of the cavitation bubble, eventually with a series of oscillations leading to a secondary cavitation bubble and so on until complete extinction on a time scale of hundreds of μs;^38,67^ and (vii) the diffusion of the laser-generated NPs in the liquid, where they can further grow, coalesce, undergo chemical reactions or be transformed by the successive laser pulses until reaching the steady-state conditions.^28,29,37,71^

How the above processes develop is determined by the experimental parameters related to the laser source (pulse duration, wavelength, repetition rate, pulse energy/fluence/power), the setup (spot size, focal length, synthesis duration, cell type, gas atmosphere), and the liquid (solvent type and solute type and concentration). Our database was built by extracting these parameters from the bibliography about the synthesis of Cu NPs by the LAL of a metal Cu target. Each LAL parameter was successively associated with one or more features (P), which are organized into four groups (G1–G4, Figure 1). G1 concerns the setup parameters: (P1) pulse duration (s), (P2) wavelength (nm), (P3) repetition rate (Hz), (P4) pulse energy (J/pulse), when available also the (P4b) fluence (J/cm^2^), (P5) lens focal length (cm), (P6) duration of synthesis (s), (P7) # of pulses of the synthesis, (P8) type of cell (1 = static cell, 2 = stirred cell, 3 = fluxed cell), and (P9) electron affinity (eV) of the elements composing the relevant gas in equilibrium with the liquid solution. G2 concerns the chemical properties of the solvent: (P10) molecular weight (MW), (P11) # of atoms in the solvent molecule, (P12) atomic % of O + Cl + CN + S in the solvent molecules, (P13) minimum bond energy (kJ/mol), (P14) average bond energy (kJ/mol) in the molecules of solvent, (P15) maximum electron affinity (eV), (P16) minimum ionization potential (eV) of molecular species expected from the fragmentation of the solvent molecules, and (P17) ionization potential (eV) of the solvent molecules. G3 concerns the physical properties of the solvent (at 25 °C): (P18) refractive index of 589 nm, (P19) relative dielectric constant, (P20) viscosity (mPa s), (P21) Henry constant (MPa), (P22) surface tension (mN/m), (P23) density (kg/m^3^), (P24) boiling point (K), (P25) melting point (K), (P26) specific heat capacity (J/mol K), (P27) thermal conductivity (W/K m), and (P28) sound speed (m/s). G4 concerns the chemical and physical properties of the most abundant solute: (P29) MW, (P30) # of atoms in the molecules of solute, (P31) % of O + Cl + CN + S in the molecules of solute, (P32) minimum bond energy (kJ/mol), (P33) average bond energy (kJ/mol) of the molecules of the solute, (P34) maximum electron affinity (eV), (P35) minimum ionization potential (eV) of molecular species expected from the fragmentation of the solute, and (P36) mass fraction of the solute in the solution. When no solutes are explicitly reported, either oxygen from the atmosphere or the inert gas bubbled in the liquid (if any) was considered as the relevant solute.^30,47,72^

In the literature about the physical–chemistry of the LAL processes, the features in G1–G4 have been used for the quantitative description of laser beam propagation in the liquid,^28,46^ laser interaction with the target or the plasma plume^28,38^ (after ca. 1 ns, the laser pulse overlaps with the plasma plume^26^), presence of compounds from the solution (solvent and solutes) in the plasma and in the cavitation bubble,^27,29,38,46^ chemical reactivity of solution species with target species, atmospheric oxygen or plasma electrons,^29,38,46,71,72^ and the physics of the cavitation bubble.^38,67,73^

On the other hand, the selection of features is bound to the data available from the literature. For instance, fluence and spot size were seldom reported, whereas pulse energy and focal length were available in all cases. Also, nonlinear refractive index, linear refractive index in the near-infrared or near UV, and acoustic impedance are not available for most liquids and were replaced with, respectively, the (linear) refractive index at 589 nm and sound speed at 25 °C. Target surface properties like roughness, purity, and contamination are generally unknown. Concerning the choice of using the % of O + Cl + CN + S in solvent and solutes, this is based on the strong empirical evidence in the literature that any of these elements can react with Cu.^20,30−35^ Hence, this feature was preferred over the % of each single chemical group (O, Cl, CN, S but also C, H, and N) to reduce the already cumbersome list of parameters and facilitate the discovery of mutual correlations with the other features whose role remains more obscure.

### General Approach

**Figure 2:**
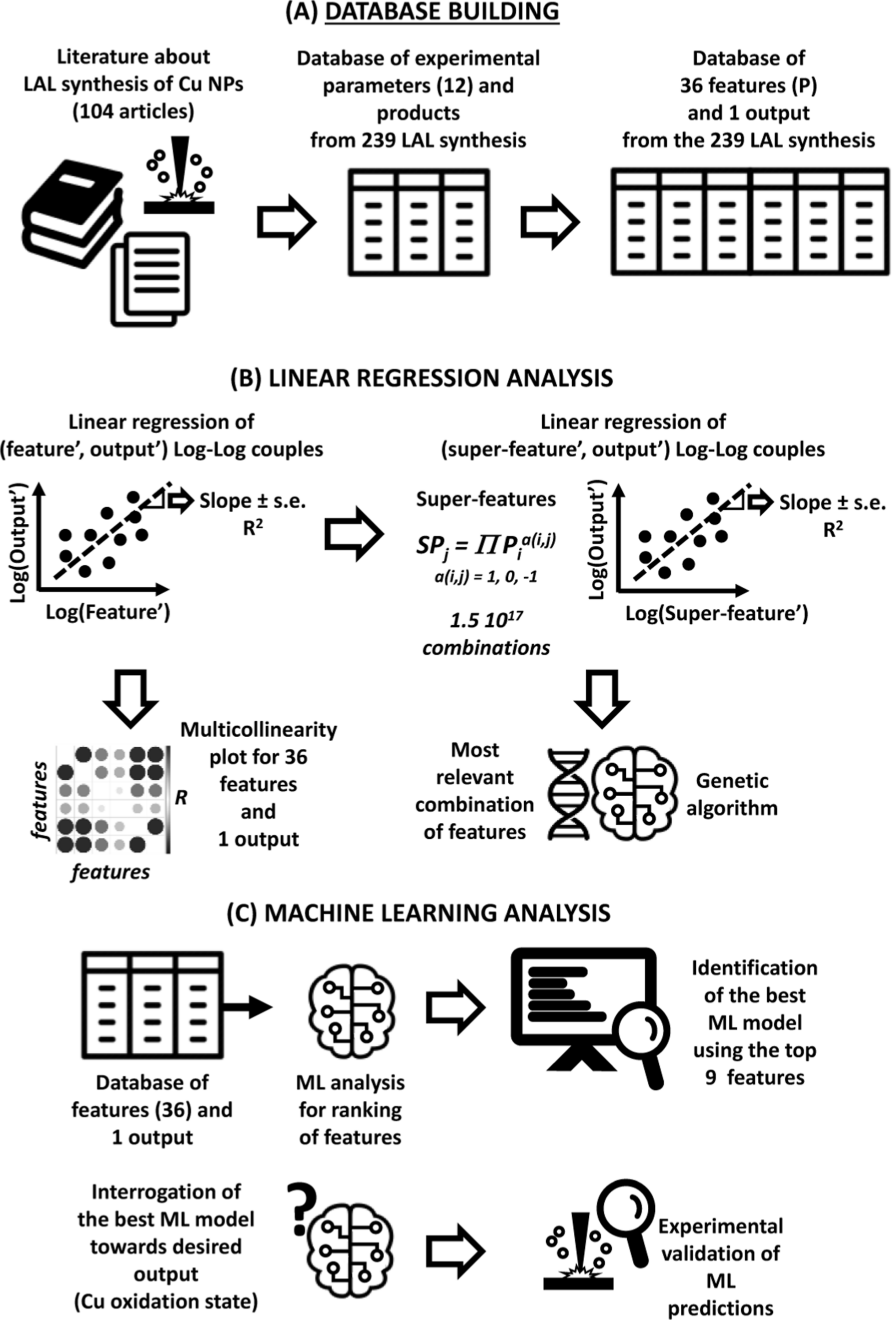
Schematic workflow of this study: (A) building of a database from the literature about LAL of Cu metal plates in liquid; (B) linear regression and GA analysis on features and their combinations (superfeatures). For the definition of feature′, superfeature′, and output′, see Section S1 in the Supporting Information; (C) ML analysis for identification of the best model and the most important features, with the prediction of experimental results and guidance to the achievement of NPs with a predetermined Cu oxidation state.

The general concept of the approach followed in this study is illustrated in Figure 2. After literature analysis and database building from 104 articles, the 36 resulting features (plus fluence, P4b) were screened for correlation and relevance to the synthesis output. The most convenient product output was identified with the average oxidation state of laser-generated Cu NPs (weighted on the relative mass of each phase). Starting with the simplest approaches, before passing to a more modern method such as the use of a genetic algorithm (GA), the linear regression analysis was applied to single features or selected groups of features (superfeatures), obtained as described later in the text and in the Section S1 in the Supporting Information. The objective was searching for a correlation with the oxidation state of the products and checking for multicollinearity, which, as GA and ML, has not been studied for the LAL synthesis to date. Subsequently, a GA was applied to delve deeper into the combination of features, which is best related to the synthesis output. Finally, the ML models were applied to the list of 36 features to disclose the cross-correlations with the corresponding output, which are hidden from the previous analysis. The ML approach started by training multiple models with the database of features to rank the features on their importance for training the models. Successively, the best-performing ML models for learning from the literature data were identified. This is the milestone to validate the ML method by comparing its predictions with LAL experimental results and guide the harvesting of Cu NPs with a predetermined oxidation state.

### Feature Analysis with Linear Regression and GA

**Figure 3:**
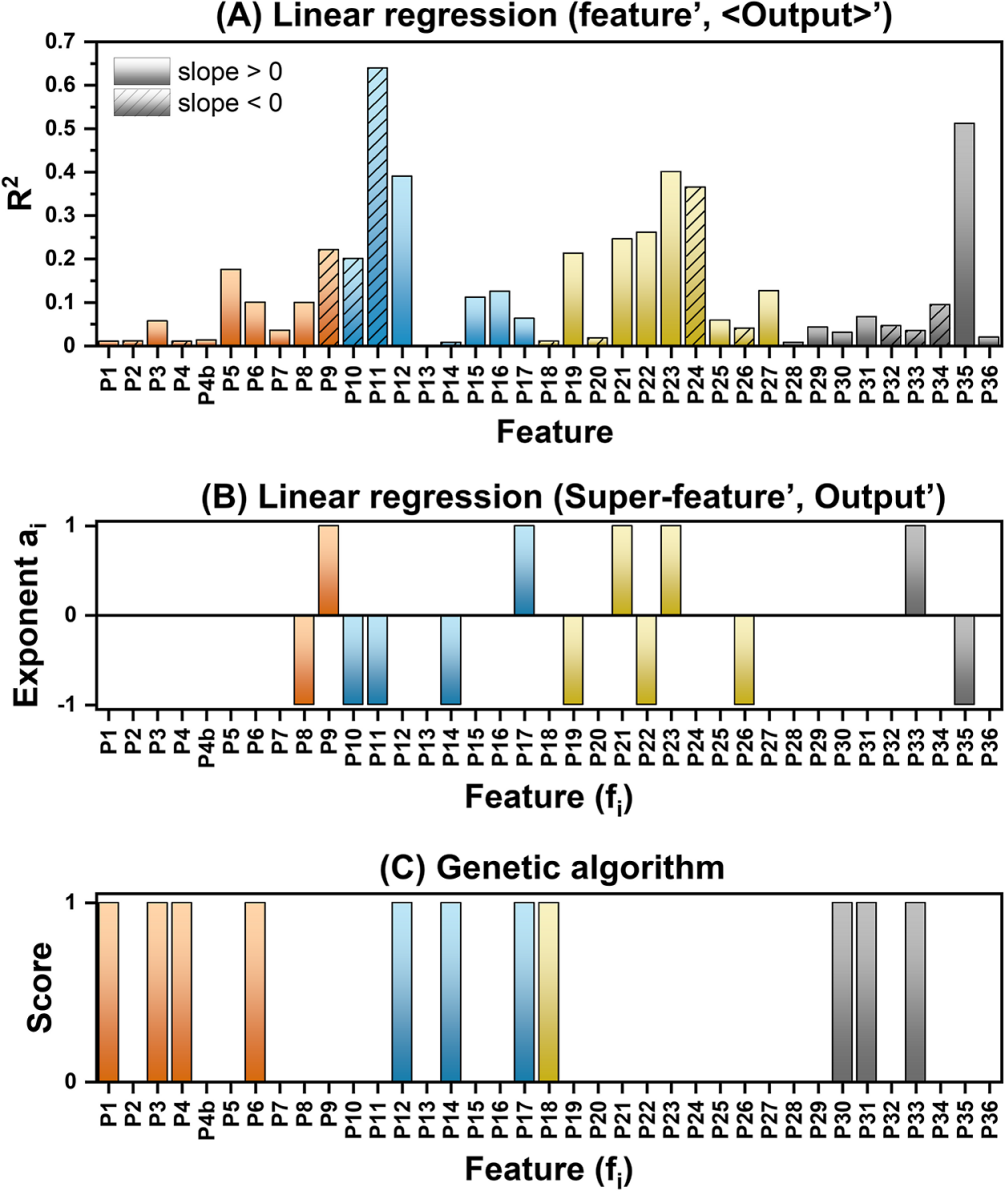
(A) *R*^2^ for the linear regression of single-feature analysis versus the average of the Cu oxidation state (⟨output⟩) for each feature value (for details, see Section S1 in the Supporting Information). (B) Exponents *a*_*i*_ (*i* = 1–36) obtained for the best superfeature SP_F, indicating the subset of 13 features required to achieve the highest *R*^2^. (C) GA score of the 36 features, identifying the subset of 11 features leading to the best linear correlation with the oxidation state of Cu.

Linear regression allows the straightforward quantitative identification of a correlation in (feature, output) data sets, providing easily understandable parameters such as the Pearson’s coefficient (*R*), the coefficient of determination (*R*^2^), and the standard error (s.e.) on the slope of the linear fit. Moreover, the sign of *R* and of the slope set the direction for changing a parameter to maximize the required output. Unfortunately, the *R*^2^ of the linear correlation between the output and each of the 36 features is always far from 1 (see Section S1 in the Supporting Information), with a maximum of 0.1647 for P12: % of O + Cl + CN + S of solvent. This is intrinsically associated with large s.e. on the slopes (Figure S2B in the Supporting Information) and indicates that this approach is not reliable for the analysis of LAL products. However, it should also be noted that some synthesis parameters are much more frequent than others in the literature used for the database. This leads to the inhomogeneous distribution of points in the features space with the accumulation of many points on the most frequent features, strongly affecting the results of the linear regression. Hence, the data sets of the average output (⟨output⟩) for each specific value in the list of a feature were also considered. The results show a general increment of the *R*^2^ (Figure 3A and Section S1 in the Supporting Information), which reached 0.6394 for P11: # of atoms of solvent molecules (s.e., 25%), 0.5115 for P35: minimum ionization potential of the solute (s.e. 27%), 0.4002 for P23: density of solvent (s.e. 35%), and 0.3898 for P12: % of O + Cl + CN + S of solvent (s.e. 38%), while being <0.38 in all other cases. By crossing the best results of the linear regression analysis, P11, P12, and P23 are the features more correlated with the oxidation state of the Cu NPs.

In reason for the overall low *R*^2^ obtained with single features, the products of features were also checked for linear correlation with the Cu oxidation state, which can be justified by a synergic effect. For instance, literature indicated that the oxidation state of Cu in NPs obtained in organic solvents may be lower when an inert gas is used to purge the liquid environment from the oxygen of ambient air,^30^ meaning that the combination of features of solvent and gas atmosphere is correlated with the average oxidation state. As the simplest approach, the superfeatures SP_*j*_ were generated as products of single features (for details see Section S1 in the Supporting Information). This approach has the limitation that the number of combinations on all the 36 features exceeded the available computational capabilities, forcing the grouping in smaller subsets. Compared to single features, the best superfeatures allowed the increment of *R*^2^ up to only 0.3122 in the best case (SP_F, s.e. = 9.6%, see Figure 3B and Section S1 in the Supporting Information), which depends on 13 features
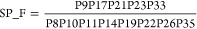
1

Noteworthily, this procedure allowed identifying a “best equation” for the prediction of the Cu oxidation state in NPs from LAL, which is
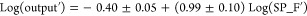
2

However, the low *R*^2^ obtained by linear regression with features and superfeatures suggests the absence of a physical or chemical reason for expecting a simple linear correlation with the oxidation states of Cu NPs as that of eq 2.

**Figure 4:**
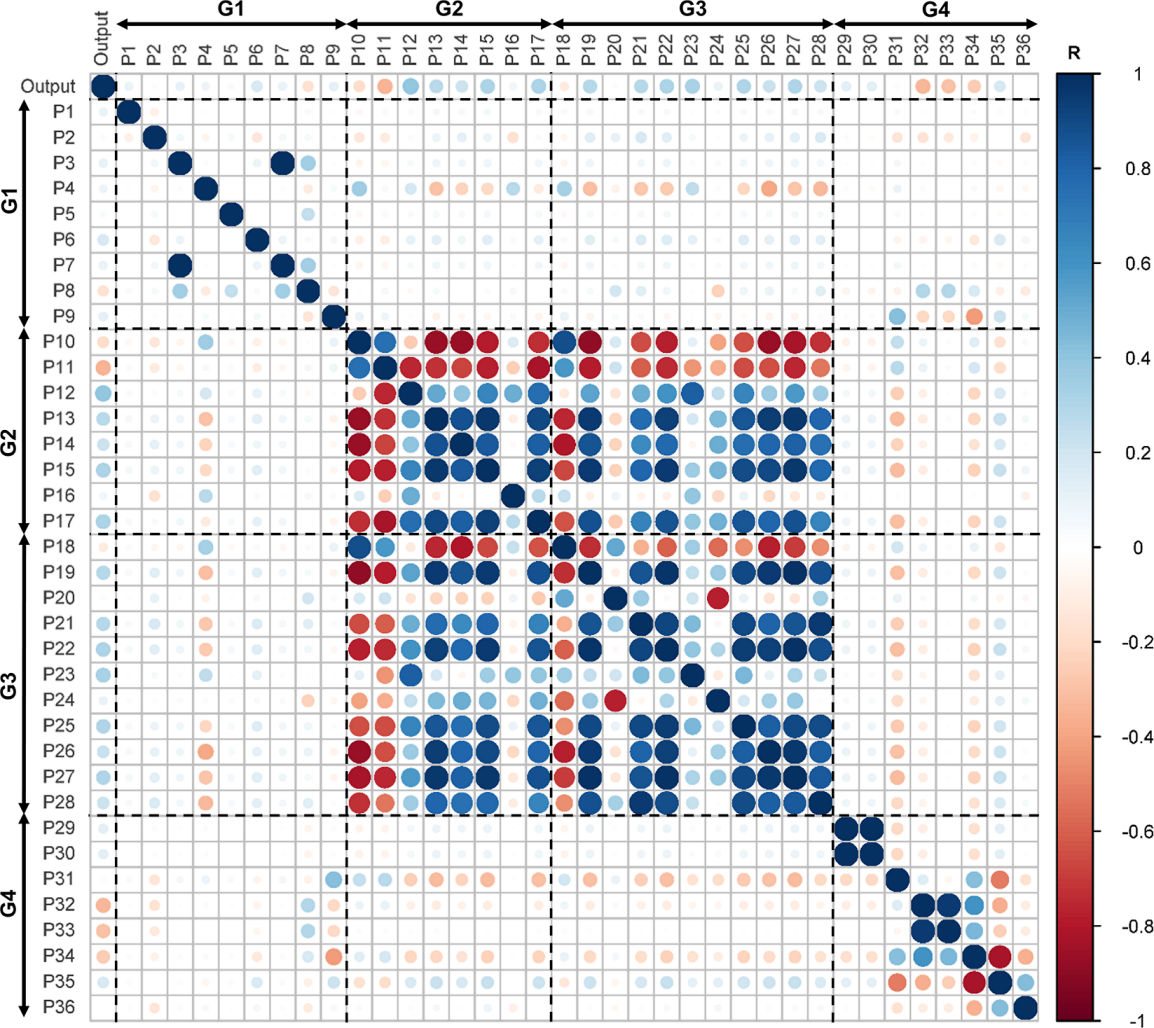
Multicollinearity plot of the 36 features and the output (Cu oxidation state). The diameter of each circle is proportional to the absolute value of *R*.

The multicollinearity plot (Figure 4) is another approach to infer the cross-correlations in multidimensional problems through transformation into a simple multivariable linear regression problem. As it is expected for the various physical and chemical properties of liquids, the plot evidenced that most features of G2 and G3 are positively or negatively cross-correlated, which also occurs for some features of G4. The features of G1 are all independent of each other, and there are no cross-correlations among the setup (G1), liquid (G2 and G3), and solute (G4) groups. This scenario pushes us to the use of a modern feature selection approach based on a GA to delve deeper into the complex cross-correlations between the features and Cu oxidation state. The GA incorporated three mechanisms (selection, crossover, and mutation) to iteratively evolve and select the most promising feature combinations linearly correlated with the output. Indeed, the resulting best *R*^2^ obtained by the GA feature selection is 0.2293, lower than 0.3122 obtained with the superfeature analysis, with a subset of 11 features over 36. The low *R*^2^ confirms further that the linear regression models are not effective in handling such a complex data set. In general, when applied to complex data sets, the effectiveness of feature selection methods based on linear regression models can heavily depend on the nature of the data and the specific problem at hand,^42,55^ and the superfeature analysis performed slightly better because the cumulative product of features introduced a higher flexibility for accounting complex interactions, which is beneficial in those cases where the relationships between the features is intricate but at the cost of reducing the interpretability of the results (13 features instead of 11), increasing the computation time and leaving unexplored a portion of the 1.5 × 10^17^ possible superfeatures.

The subset of 11 features identified with the GA are (P1) pulse duration, (P3) repetition rate, (P4) pulse energy, (P6) duration of synthesis, (P12) % of O + Cl + CN + S, (P14) average bond energy, (P17) ionization potential, (P18) refractive index at 589 nm, (P30) # of atoms, (P31) % of O + Cl + CN + S, and (P33) average bond energy. These 11 features are different, except P14, P17, and P33, from those identified by the superfeature analysis and only have P12 in common with the best results of the single-feature analysis versus the average output (Figure 3B,C).

### ML Analysis

**Figure 5:**
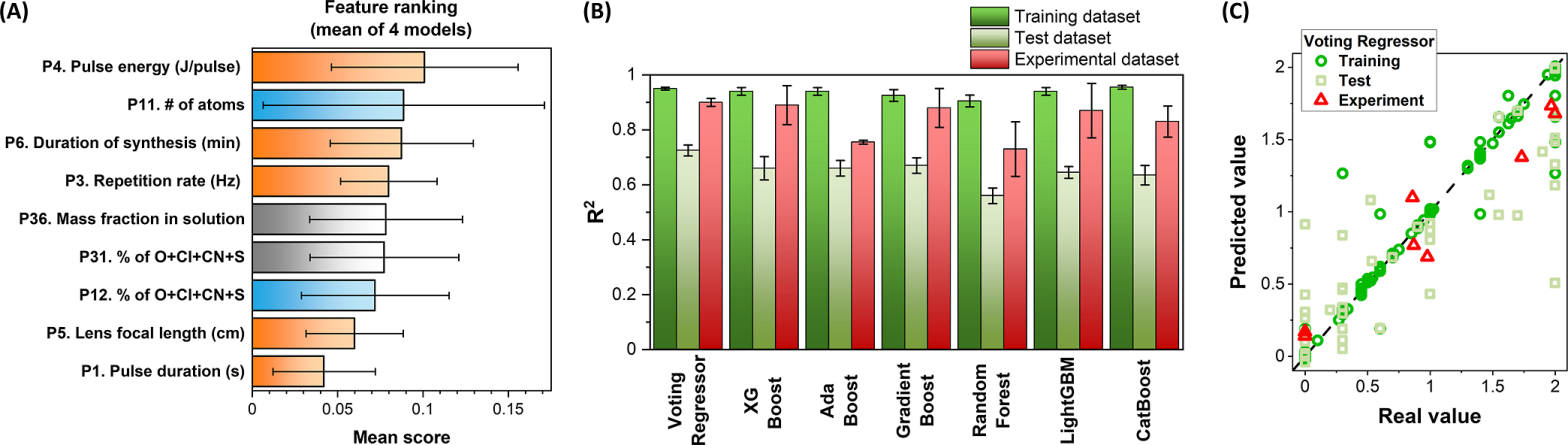
(A) Top nine features according to the average score obtained in the ranking procedure with XG Boost, Ada Boost, Gradient Boost, and Random Forest models. (B) *R*^2^ of the linear fit of the predicted values versus real values for the training and test data sets taken from the literature. Red bars report the *R*^2^ of the linear fit for the predicted values versus the values obtained from the experiments in this study. (C) Plot of predicted values versus real values for the training, test, and experimental data sets using the voting regressor model. The dotted line indicates a perfect correlation (*R*^2^ = 1).

Considering the previous results, we resorted to the superior flexibility of the ML approach to unravel the complex cross-correlations between the features which are relevant for determining LAL products and were not accessible with the linear regression analysis through the three different approaches tested (GA, single features, and superfeatures). Several ML models were screened for their ability to learn the nonlinear relationships between the input conditions (features) and the predicted properties (outputs), based on the previous literature evidencing their success in designing synthetic pathways from data sets with a size of a few hundreds.^42,51,54,55,74,75^ The main ML approaches considered in these preliminary tests included supervised learning and supervised ensemble methods based on boosting or bagging.^42,55^ This led to the selection of six regression models (XGBoost, AdaBoost, GradientBoost, Random Forest, LightGBM, and CatBoost)^42,55^ and to the exclusion of artificial neural networks which, in our tests, systematically performed worse (see Table S3 in the Supporting Information). Then, the features were ranked through the permutation-based feature importance method built in four ML models (XGBoost, AdaBoost, GradientBoost, and Random Forest). Considering the difficulties evidenced with high-dimensional data sets by feature selection approaches like the superfeature analysis and GA, in this case, a permutation-based feature importance ranking method and robust tree-based models were adopted, which excel in handling complex relationships within the data. The ranking led to only nine features with an average score >0.04 (Figure 5A and Table S4 in the Supporting Information). Five features are from the G1 about setup parameters (P4: pulse energy, P6: duration of synthesis, P3: repetition rate, P5: lens focal length, and P1: pulse duration), two from the G2 about the chemical properties of liquid (P11: number of atoms in solvent molecules and P12: % of O + Cl + CN + S in solvent molecules), and two from the G4 about the properties of the prevailing solute (P31: % of O + Cl + CN + S in solute molecules and P36: mass fraction of the solute in the solution). The 10th and 11th features are from G3 about the physical properties of the solvent (P18: refractive index at 589 nm and P24: boiling point), but they were excluded from the next step due to the lower score and the purpose of limiting the number of features as much as possible to avoid the model performance decrease.^42,55^ Then, the data set was split into 80% of training set and 20% of testing set, and the 5-fold grid-search cross-validation (GridSearchCV) method and Bayesian optimization method^42,55,76^ were utilized to tune the hyperparameters of six models to obtain the optimal fitting performance starting from the nine best features. Under optimal hyperparameter settings, all models performed very well on the training data set, but only some of them reached satisfactory performance also on the test data set (Figure 5B).

The general homogeneity of the performance throughout models based on different approaches such as boosting (AdaBoost, XGBoost, Gradient Boost) or bagging (Random Forest)^42,55^ substantiate the reliability of this procedure. However, considering the small size of the database, overfitting may take place independently of the selected hyperparameters for all models. This problem was mitigated by resorting to the voting regressor model,^42,55^ which averages the individual predictions of a set of ML models by the voting mechanism. The XGBoost, AdaBoost, GradientBoost, LightGBM, and CatBoost boosting models were used as base estimators of the voting regressor trained with the same data set and features. With the voting regressor model, the *R*^2^ of the linear fit for predicted versus real values resulted in 0.95 for the training set and 0.72 for the test set (Figure 5B,C). The trends of the mean absolute error (MAE) and the root-mean-square error (RMSE) follow that of *R*^2^, confirming the best performance of the voting regressor.

In order to verify the optimal dimensionality of the descriptors for this database, the hyperparameters of the ML models were reoptimized also by including the 10th and 11th feature from the ranking of Table S4. The performance remained stable or slightly worsened with 10 and 11 features (see Section S2 in the Supporting Information), indicating that the increase of the number of features is unnecessary and not insightful about the synthesis process.

Finally, to account for the generally different accuracy in product assessment from different sources and to improve the statistical validity of the method, the ML model was operated by selecting the training and test data sets from different articles, i.e., by avoiding the case where the articles describing more than one LAL condition contribute simultaneously to the training and test data sets. In this test, the hyperparameters and the number of features remained the same as in the unsplit database. Despite the small size of the database, the voting regressor model remained robust enough to maintain similar performances even in this less favorable condition (see Section S3 in the Supporting Information).

### ML Predictions and Guiding of Synthesis Conditions

The voting regressor can be exploited to obtain useful indications for the synthesis of NPs with the desired Cu oxidation state under the typical experimental conditions of a specific laboratory. In our case, the typical LAL conditions allowed us to fix the five features from G1 (P1: 6 × 10^–9^ s; P3: 50 Hz; P4: 0.05 J/pulse; P5: 10 cm; and P6: 180 min) and investigate the role of the remaining four features from the solvent and solute. Indeed, the prevalence of setup features (5 over 9) is an advantage to simplify the predictions of synthetic conditions in a typical LAL laboratory condition.

**Figure 6:**
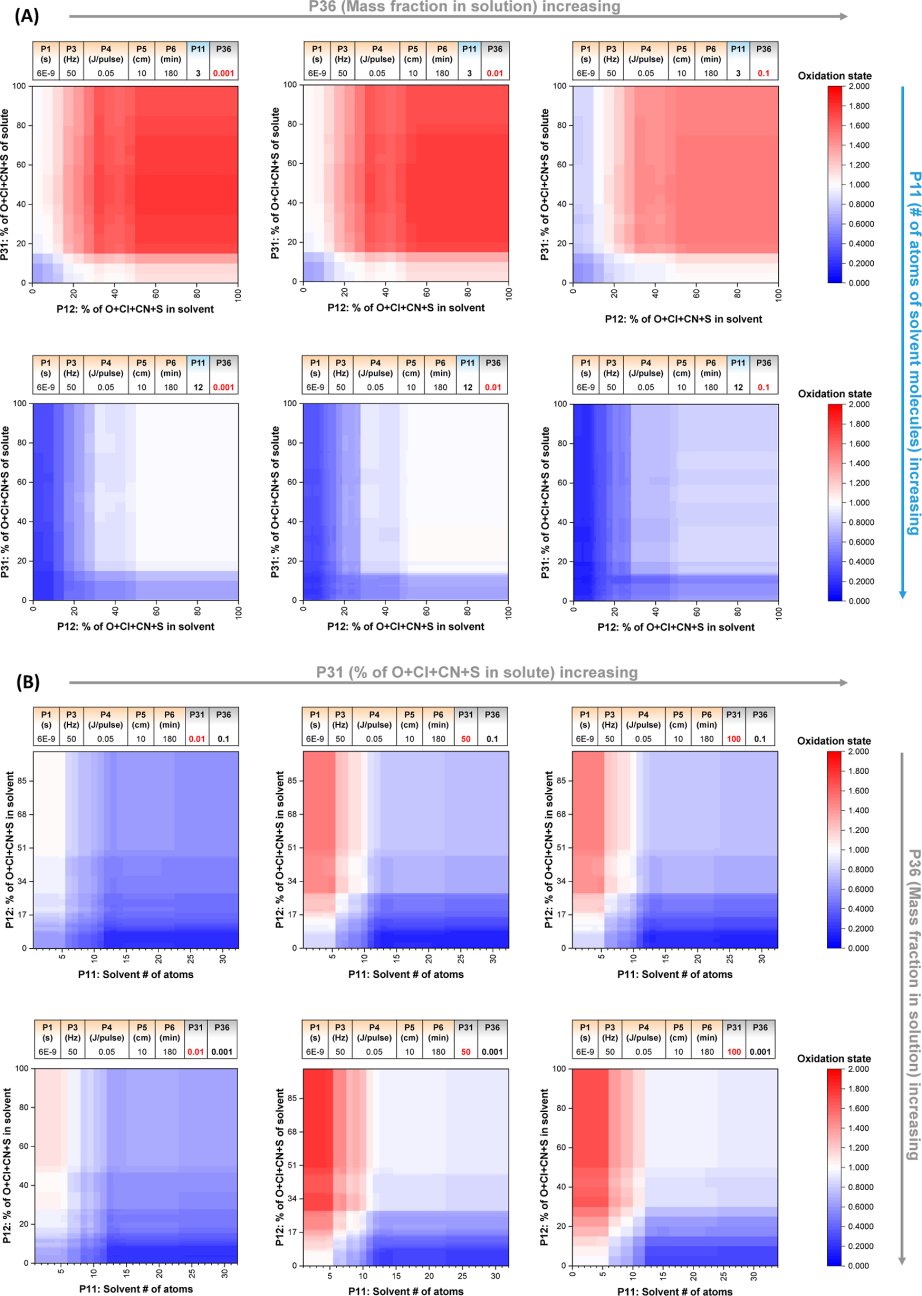
(A) Prediction of the variation of the oxidation state as a function of the % of O + Cl + CN + S in solvent molecules (P12) and solute molecules (P31) at three solute concentrations (P36 = 0.001, 0.01, and 0.1) and two different numbers of atoms in solvent molecules (P11: 3 and 12). (B) Prediction of the variation of the oxidation state as a function of the number of atoms (P11) and of the % of O + Cl + CN + S (P12) in solvent molecules, at three % of O + Cl + CN + S in solute (P31 = 0.01, 50, and 100) and two solute concentrations (P36 = 0.001 and 0.1). Setup parameters are fixed as reported in the tables above each graph. All predictions are obtained with the best model (voting regressor).

Initially, the effect of the % of O + Cl + CN + S in the solvent molecules (P12) and solute molecules (P31) on the oxidation state has been predicted for three solute mass fractions (P36 = 0.001, 0.01, and 0.1) and solvent molecules with a different number of atoms (P11: 3 and 12), as shown in Figure 6A. For small solvent molecules (3 atoms), the general trend shows a trade-off around oxidation state +1 when both the % of O + Cl + CN + S in solute and solvent increase above 10–20% (variable with the solute mass fraction). However, the intervals for achieving the oxidation state +1 (white regions) are tight at all concentrations of solute, and they shift to higher values of P12 when P36 also increases. The scenario is different for solvent molecules with 12 atoms, for which there is a wide range allowing the oxidation state +1, provided that the mass fraction of the solute is below 0.01 and the % of O + Cl + CN + S differs from 0 in the solvent and solute, as happens for alcohols.

Given the relevance of the number of atoms (P11), its effect versus the % of O + Cl + CN + S (P12) in solvent molecules was plotted for three % of O + Cl + CN + S in the solute (P31 = 0.01, 50, and 100) and two solute concentrations (P36 = 0.001 and 0.1), as shown in Figure 6B. The number of atoms in the database ranged from 3 (water) to 32 (decane). For solutes with a negligible % of O + Cl + CN + S (P31 = 0.01), the oxidation state < 1 prevails when P11 increases above 5. When P31 is 50 or 100, the oxidation state > 1 becomes possible at a high % of O + Cl + CN + S in the solvent (P12) and a number of atoms < 5. Again, there is a tight area in which the oxidation state +1 is predicted in all cases except for small solvent molecules (P11 < 5) and appreciable % of O + Cl + CN + S (P12 > 30%) but in the presence of a relatively high mass fraction of solute (P36 = 0.1) without oxygen (P31 = 0.01). These conditions are not easy to be achieved, considering that in general, nonpolar solutes cannot be dissolved at high concentrations in polar solvents. However, by coupling the information from the plots in Figure 6A,B, one can infer that LAL in water (P11 = 3, P12 = 33%) with solutes like alcohols (P31 = 8–11%) or Ar (no oxygen, P31 = 0%) is expected to provide NPs with a Cu oxidation state of +1. Similarly, acetonitrile (P11 = 6, P12 = 33%) with Ar (P31 = 0%) is on the edge of the region for oxidation state +1 in the (P11, P12) plots, and the LAL products obtained in this environment should have a Cu oxidation state close to +1 as well. Conversely, resorting to oxygen-rich solvents and solutes will shift the Cu oxidation state well above +1 and, on the contrary, oxygen-poor solvents and solutes will keep the oxidation state close to 0.

**Figure 7:**
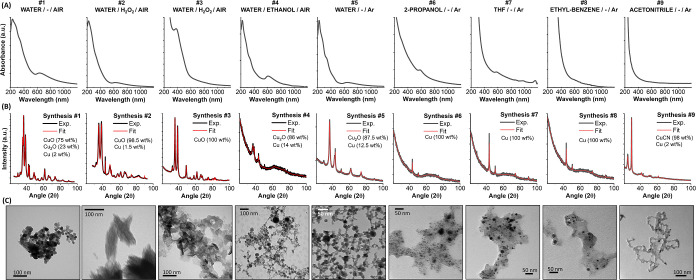
Experimental data about LAL of Cu-based NPs using the setup conditions described in Figure 6 and with various combinations of solvent and solute. (A) UV–vis spectroscopy of the colloid. (B) XRD analysis and Rietveld refinement. (C) Transmission electron microscopy (TEM) analysis.

Hence, a series of LAL experiments were performed (see Figure 7 and Table 1) to cover this set of combinations of solvent and solute parameters and seek the various Cu oxidation states. The results, summarized in Table 1, are in fair agreement with the predictions of the voting regressor ML model with an *R*^2^ of 0.90 (red triangles in Figure 5C), confirming the reliability of the overall procedure and the physical–chemical insights derived from it.

**Table 1:** Summary of the LAL Experiments^a^

id	solvent	solute	gas	products	average oxidation state (experiment)	oxidation state (ML prediction)
#1	water		air	CuO (75 wt %)–Cu_2_O (23 wt %)–Cu (2 wt %)	1.73	1.38
#2	water	H_2_O_2_ (0.1 wt %)	air	CuO (98.5 wt %)–Cu (1.5 wt %)	1.97	1.73
#3	water	H_2_O_2_ (5.5 wt %)	air	CuO (100 wt %)	2.00	1.68
#4	water	ethanol (5.3 wt %)	air	Cu_2_O (86 wt %)–Cu (14 wt %)	0.86	1.10
#5	water		Ar	Cu_2_O (87.5 wt %)–Cu (12.5 wt %)	0.88	0.77
#6	2-propanol		Ar	Cu (100 wt %)	0.00	0.14
#7	tetrahydrofuran		Ar	Cu (100 wt %)	0.00	0.14
#8	ethyl benzene		Ar	Cu (100 wt %)	0.00	0.17
#9	acetonitrile		Ar	CuCN (98 wt %)–Cu (2 wt %)	0.98	0.69

a Laser and setup parameters are in all cases 1064 nm, 6 ns, 50 Hz, 50 mJ/pulse, 6.4 J/cm^2^, focal 10 cm, static cell, 360 min (#1–#4), or 180 min (#5–#9).

## Discussion

**Figure 8:**
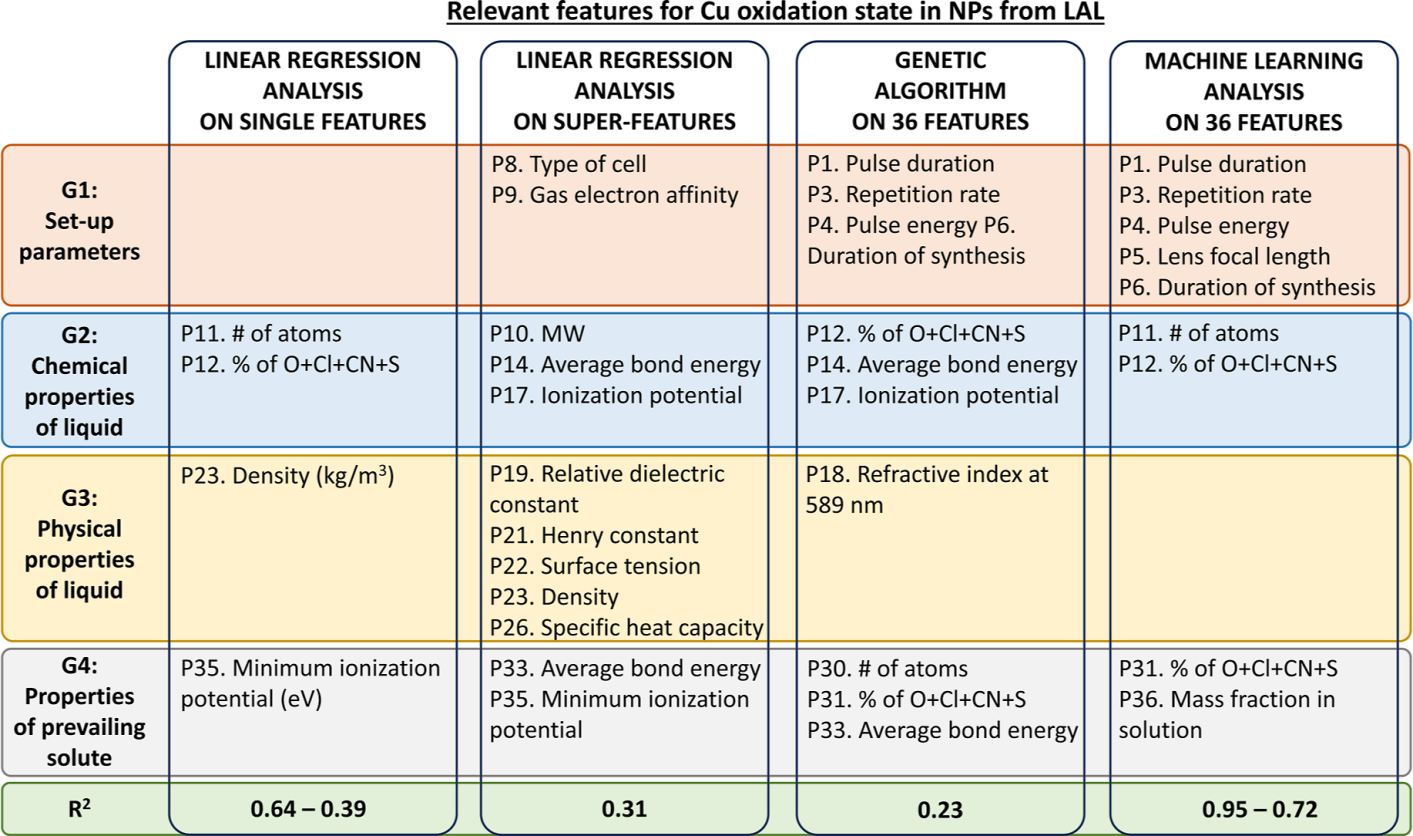
Summary of the most relevant features for the determination of Cu oxidation state in copper-based NPs obtained by LAL, according to the linear regression, GA, and ML analyses.

Cu-based NPs are relevant components for several advanced technologies integrated with renewable energy sources, and the identification of sustainable production processes is key to their successful exploitation. LAL is a viable candidate to produce Cu-based NPs, but it is still difficult to predict and optimize the products among the variety of compounds and oxidation states that Cu can form. The analysis of the database of LAL synthetic conditions potentially contains the required information for identifying the relevant features connected to the synthesis, as summarized in Figure 8, which can guide the experimental activity toward harvesting the desired Cu product. However, the literature does not provide any indication about the most suitable mathematical model or algorithm for this type of predictions.

In the simplest approach, each single feature was analyzed through a linear regression approach. This method indicated that the oxidation state of Cu has the highest correlation with the % of O + Cl + CN + S, the # of atoms of solvent molecules, and the density of solvent (see Section S1 and Figure S2 in the Supporting Information). This result agrees with the empirical observation that O, Cl, S, and CN react with copper giving nonmetallic NPs, explaining the positive slope. Moreover, the % of these functionalities in the molecules is usually lower when the number of atoms increases (typically, this corresponds to alkanes and their derivatives in the literature used for the database), explaining the negative slope, while the % of O + Cl + CN + S is higher when the density increases (for instance, water, chloroform, dichloromethane, acetonitrile, and dimethyl sulfoxide), explaining the positive slope.

Since these considerations add little to the empirical observations, an attempt to unravel the crossed correlations between the features was done with superfeatures and the GA. The linear regression analysis of superfeatures suggested that complex relations among features and the oxidation state of Cu exist. However, the results add little to the interpretation of the fundamental aspects of the process and are of little help in guiding the synthetic effort in ordinary laboratory conditions. Most importantly, the *R*^2^ is low, meaning that the prediction is inaccurate; in fact, the data set is distributed in three groups in the plane of Log(output′) versus Log(SP_F′) (see Section S1 and Figure S4 in the Supporting Information), which indicates that the relation between features and the oxidation state cannot be described with this simplistic approach. The GA did not perform better than the superfeature analysis, and the two subsets of features only have three features in common.

This prompted the application and screening of the ML models, which are renowned for their superior flexibility and ability in catching complex and hidden relations among features toward a certain output. The first result produced by the ML analysis consisted of a ranking of features relevant to the prediction of the output (Figure 5A). Of the 9 most relevant features, five describe the experimental setup (P4: pulse energy, P6: duration of synthesis, P3: repetition rate, P5: lens focal length, and P1: pulse duration), while only two describe the chemical properties of the solvent (P11: number of atoms in the solvent molecules and P12: % of O + Cl + CN + S in the solvent molecules), and two describe the properties of the most abundant solute (P31: % of O + Cl + CN + S in the solute molecules and P36: mass fraction of the solute in the solution). The presence of P11, P12, P31, and P36, which are connected to the chemical properties and concentration of the liquid solution, is very reasonable based on the experimental intuition and the *R*^2^ of the previous linear regression analysis. More surprising is the presence of setup features P1, P3, P4, P5, and P6, which systematically exhibited low *R*^2^ in the linear regression analysis, although P1, P3, and P4 were also selected by the GA. This is indicative of the setup features having a nontrivial effect on the chemistry of the nanomaterials generated by the laser synthesis, which is in general agreement with a long list of studies observing different chemical compositions and NP formation mechanisms by acting on these parameters.^27−30,32,38,47,71,72^

Solvent physical properties have a lower relevance for the identification of nonlinear correlations between the features and the oxidation state of copper in NPs by the ML models. Noticeably, the performance of the ML models remained stable or slightly decreased when the 10th and 11th features, both from G3 (P18: refractive index at 589 nm, P24: boiling point), were also added (Section S2 in the Supporting Information), as it is typical of ML models.^42,55^

Hence, the great advantage brought by the ML analysis is in the drastic reduction of synthesis parameters for predicting the oxidation state of Cu, especially in the case of a predetermined setup. The best ML model allowed for identifying the maps of the oxidation state versus solvent and solute chemical composition, solvent molecular structure, and solute concentration (Figure 6). The analysis of these maps is simple and straightforward for identifying the combination of parameters leading to a predetermined oxidation state, even for the intermediate Cu(I) compounds, which have the tightest permitted region.

The experimental validation of these predictions was performed to demonstrate the utility of the ML approach for guiding the real synthetic operations and show how it can be integrated with the common experimental practice, which is usually limited to a collection of experimental results such as those in Figure 7. Following the maps of Figure 6, Cu NPs with various average oxidation states were produced, including the oxidation state close to 1, which has a tight range of existence, in three cases (Table 1). It also confirmed that the % of O + Cl + CN + S in the solvent and solute molecules are crucial for the chemical composition of LAL-generated Cu NPs, but these parameters should be tuned in agreement with solute concentration and solvent structure (number of atoms) to achieve an accurate control on the products. With the ML model, the oxidation state of Cu can also be predicted in other experimental conditions relevant for high-throughput LAL synthesis of NPs (see Section S4 in the Supporting Information), which requires kHz or MHz repetition rates with ns or picosecond pulses with energy in the range of tens of μJ. However, there are less data available in literature for these experimental conditions, which currently represent one of the frontiers for the future development of this approach. Indeed, since the LAL synthesis is very well suited to the creation of large databases of features and outputs, there is significant room for future improvements, such as the prediction of the specific compound and NP size as well as the application to the whole library of nanomaterials accessible by laser synthesis and processing, starting from the milestone represented by this work.

## Conclusions

This study contributed to the urgent need for sustainable synthetic processes for Cu-based nanocrystals in the greatest demand for integration in emerging green technologies. The LAL synthesis was analyzed with linear regression and ML models to unravel the complex relations between the multiple features of the experimental procedure toward the achievement of the desired Cu oxidation state. A database containing 36 features was built from the literature, and the linear regression analysis was initially applied to confirm the importance of solvent (% of O + Cl + CN + S, # of atoms of solvent molecules, density). An approach based on combinations of the features (superfeatures) led to an equation describing the main features involved in the determination of the Cu oxidation state (type of cell, gas electron affinity, solvent MW, number of atoms, average bond energy, ionization potential, relative dielectric constant, Henry constant, surface tension, density, specific heat capacity, solute average bond energy and minimum ionization potential). Nonetheless, the superfeature resulted in complex interpretation and application and of low accuracy. Overall, the linear regression analysis was inadequate to understand the cross-correlations between the features and the chemistry of the LAL products, even when implemented in a GA.

Hence, the ML approach was adopted, unveiling unexpected correlations between setup features (pulse energy, duration of synthesis, repetition rate, lens focal length, and pulse duration), solvent chemical composition (# of atoms and % of O + Cl + CN + S in the molecules), and solute parameters (% of O + Cl + CN + S and mass fraction). The best ML model resulted in great efficacy and practical utility in identifying the synthesis pathway toward a given oxidation state, starting from a specific setup. Guided by the ML maps, new experiments were performed for targeting the various oxidation states of Cu, including the challenging Cu(I) compounds, which can be obtained only within restricted intervals of the experimental features. The agreement between the experiment and ML predictions resulted in an *R*^2^ of 0.9, leading to the identification of three different sets of experimental conditions yielding Cu-based NPs with a copper oxidation state close to 1. This further expands the versatility of LAL for the generation of Cu-based nanocrystals of great interest for integration in sustainable processes ranging from electrocatalysis to photocatalysis, photovoltaic cells, and many others. In addition, the ML approach is of general applicability to other nanomaterials and opens new perspectives for understanding the origin of the chemical pathways of nanomaterials generated by LAL. Thus, departing from this milestone, we expect that the laser synthesis and processing of colloids will be empowered with a rational guideline for the conscious predetermination of synthetic parameters toward the desired compound among the huge library of nanomaterials.

## Methods

### Data Set

The data set consists of 239 LAL experiments extracted from 104 scientific articles about LAL of metal Cu targets published until 31st July 2022. The list is provided as an Excel file in the Supporting Information (source data Tables S8–S13). The list of articles was maintained unchanged over time to provide a fixed data set for the regression and ML analysis. The information on synthesis conditions extracted directly from the articles, wherever available, includes laser pulse duration, wavelength, repetition rate, pulse energy, fluency, power or intensity, spot size or area, lens focal length, synthesis duration or number of pulses, cell type, gas atmosphere, solvent, solutes, and their concentration. For products, the relevant information extracted from the articles includes NP phases, relative quantity, and experimental technique used for their assessment. The database obtained after the necessary data cleaning for fixing the anomalies, such as incomplete or duplicate records,^52^ is provided as an Excel file in the Supporting Information (Tables S8–S13).

As is typical of experimental data taken from the literature, the experimental methods and the number of experimental techniques adopted change significantly from article to article, which introduces an unavoidable variability in the reliability of the data. In particular, the assessment of the composition with XRD and the Rietveld refinement of the diffractogram are the most reliable experimental methods to quantitatively identify the composition of the samples, but this method was used only in a minority of the studies. Since the limited size of the database does not allow reducing it to the reports with XRD data without significantly affecting the ML performance, this is a relevant hidden variable and a possible source of inaccuracy in the input data. For tracking this issue, the experimental techniques used for the assessment of the composition have been provided in Table S8 in the Supporting Information.

### Linear Regression Analysis and GA

In linear regression analysis, a home-built code was used for the adaptation of (feature, output) or (superfeature, output) data from our database to the Log–Log plot (Log = log_10_), as described in Section S1 and Figure S1 in the Supporting Information, leading to the final (feature′, output′) or (superfeature′, output′) data sets. The shifted data sets were used for the linear regression, extracting the coefficient of determination, *R*^2^ (or *R*-squared), the slope, and the standard error on the slope for each feature or superfeature.

The multicollinearity of the 36 features and the output were obtained and plotted with a standard python routine.

The GA was based on three primary mechanisms: selection, crossover, and mutation, to iteratively evolve and select the most promising feature combinations to describe the output. Each individual in the population is represented as a binary string, encoding a specific subset of the 36 features.^42,55^ The *k*-fold cross-validation was employed to evaluate the fitness of each individual. This enables a reasonable assessment of the individual’s performance on diverse data partitions, enhancing the overall reliability of our results and ensuring the robustness of the approach. The GA was designed to optimize two critical fitness criteria simultaneously, using linear regression as the base model: maximization of the coefficient of determination (*R*^2^) and minimization of the MSE.

### ML Analysis

In ML analysis, the Python scikit-learn packages 1.2.2^77^ and XGBoost package 1.7.0 were used to identify the most suitable models for nonlinear regression feature selection among XGBoost, Ada Boost, Gradient Boost, Random Forest, Decision Tree Regressor, and Lasso regression. *R*^2^ was used to evaluate the goodness-of-fit of each model; MAE and RMSE were calculated for a further comparison of the performance of different models and verification of the most appropriate one.^42,55^ The raw data set with 36 features was input into the six models to obtain the *R*^2^ score under default hyperparameter settings. The resulting *R*^2^ ranking was XGBoost > Gradient Boost > Ada Boost > Random Forest > Decision Tree > Lasso regression. According to this ranking, the permutation-based feature importance method built in ML models was used to get the importance scores of the 36 features for the top 4 models (XGBoost, GradientBoost, AdaBoost, and Random Forest). In this feature ranking procedure, a permutation-based feature importance ranking method and robust tree-based models were applied. Hence, all of the feature values were randomly shuffled to destroy the information existing in each input and automatically compute the variation of model’s performance, so that the predictive usefulness of each input feature can be determined. Subsequently, the hyperparameters of XGBoost, Ada Boost, Gradient Boost, Random Forest, Decision Tree Regressor, and Lasso regression models were optimized by utilizing the 5-fold grid-search cross-validation (GridSearch CV) method in the scikit-learn package. Given a large search range and a small step size, the grid search method is certain to find the global maximum or minimum. Usually, the grid search method is initially used with a larger search range and larger step size to identify potential global extrema because of its heavy computational resource consumption, especially when dealing with multiple hyperparameters. Later, the search is refined by narrowing down the range and step size to obtain more precise optimal values. However, this refinement process may still miss the global extrema since the objective parameter is typically nonconvex.^42,55,78^ Moreover, this method led to an exponential rise in run time and computation efforts, while the size of hyperparameter space increased. Hence, this problem was mitigated by resorting to Bayesian optimization, which considers each hyperparameter set as an independent individuality. Instead of monotonously going through every hyperparameter set at random, the Bayesian optimization method can converge to the optimal hyperparameters by learning from previous iterations, with a great reduction of the computation time and elevating the probability of finding the optimal hyperparameter combination without traversing the entire search space. Bayesian optimization is also useful for model predictions in regions where sufficient training data are not available for a typical ML process.

XGBoost, Ada Boost, Gradient Boost, Random Forest, and two additional models (LightGBM and CatBoost) selected for fast execution along with the ability to maintain good performance under Bayesian optimization were employed. Finally, the XGBoost, Ada Boost, Gradient Boost, LightGBM, and CatBoost boosting models were used to form the base ensemble for the voting regressor model, which utilized weighted averages according to the base models’ performance. The procedure was performed with various combinations of test and training data sets by retaining the best two results for each model, from which a tolerance on the model performance end point (*R*^2^) was obtained (error bars in Figure 5B).

Source data and source codes are available from https://doi.org/10.5281/zenodo.8433919.

### Synthesis and Characterization

LAL was performed with 1064 nm laser pulses (6 ns, 50 Hz, 50 mJ/pulse) of a Q-switched laser focused with an *f* = 100 mm lens to a fluence of 6.4 J/cm^2^ on a 99.99% pure Cu plate (Sigma-Aldrich) located in a batch chamber filled with the liquid solutions indicated in Table 1. 2-Propanol (ACS reagent, puriss.), tetrahydrofuran (HPLC), acetonitrile (HPLC grade), and ethyl benzene (puriss.) were purchased from Merck/Sigma-Aldrich. Bidistilled water was produced in-house with a homemade bidistiller. The cell was mounted on a motorized XY scanning stage (Standa) managed with a 2-axis stepper and a DC motor controller to ablate the target along an Archimedean spiral pattern. An Ar atmosphere was obtained by bubbling the gas at constant flow in the liquid through a Teflon tube inserted in the cell cover, already 15′ before each synthesis.

UV–visible spectra after LAL were recorded with a JASCO V770 spectrophotometer using quartz cells with a 2 mm optical path. TEM analysis was performed with an FEI Tecnai G2 12 transmission electron microscope operating at 100 kV and equipped with a TVIPS CCD camera. The samples for TEM analysis were prepared at room temperature by evaporating the NP suspensions on a copper grid coated with an amorphous carbon film.

XRD patterns were collected on a Bruker D8 ADVANCE Plus diffractometer operated at 40 kV and 40 mA using a Cu Kα radiation source. The crystallographic phase identification was performed by a search/match procedure using Bruker DIFFRAC.EVA software and the COD database, while the diffractograms were analyzed with TOPAS Academic V6 (Bruker AXS). Rietveld refinements were carried out by fitting the background with a Chebychev function, a broad Gaussian peak due to the amorphous phase, and the required phases (Cu COD 9012043, Cu_2_O COD 9007497, CuO COD 7212242, and CuCN COD 1100000). The shape of the reflections was modeled through the fundamental parameter approach incorporated in the program, separating the instrumental and the sample contributions. Fit indicators *R*_wp_, *R*_exp_, and GoF (goodness-of-fit) were used to assess the quality of the refined structural models.

## Supporting Information Available

Single-parameter linear regression analysis (features and superfeatures); comparison of the ML performances with the top 9, 10, and 11 features; validation of the ML model with data set splitting for different sources; prediction of Cu oxidation state in high-throughput LAL conditions; and link to the source data and source codes (PDF)
Results with artificial neural networks and linear regression models; ranking of features from four ML models; hyperparameters of the best ML models using 9 features; hyperparameters of the best ML models using 10 features; and hyperparameters of the best ML models using 11 features (XLSX)
