# Supplementary material for: Data-Driven Predetermination of Cu Oxidation State in Copper Nanoparticles: Application to the Synthesis by Laser Ablation in Liquid
Source: J Am Chem Soc. 2023 Oct 31;145(47):25737–52. doi: 10.1021/jacs.3c09158 (PMC10690790; doi:10.1021/jacs.3c09158)
Supplement: Supplementary file 1 — ja3c09158_si_001.pdf [file ja3c09158_si_001.pdf]

Supporting Information

Data-driven pre-determination of Cu oxidation state in copper nanoparticles: application to the synthesis by laser ablation in liquid

Runpeng Miao, Michael Bissoli, Andrea Basagni, Ester Marotta, Stefano Corni, Vincenzo Amendola*

Department of Chemical Sciences, University of Padova, 35131 Padova, Italy

Email: vincenzo.amendola@unipd.it

Contents:

In this file:

S1. Single-parameter linear regression analysis (features and super-features)

S2. Comparison of the ML performances with the top 9, 10 and 11 features

S3. Validation of the ML model with dataset splitting for different sources

S4. Prediction of Cu oxidation state in high throughput LAL conditions

In the file "Supporting_Information_Tables S3-7.xlsx"

- **Table S3:** Results with Artificial Neural Networks
- **Table S4:** Ranking of features from four ML models
- **Table S5:** Hyperparameters of the best ML models using 9 features
- **Table S6:** Hyperparameters of the best ML models using 10 features
- **Table S7:** Hyperparameters of the best ML models using 11 features

***Available from* https://doi.org/10.5281/zenodo.8433919**

- **Source_Data_Tables_S8-S13.xlsx:** Database of literature data about LAL of Cu and relative features
- **Source codes:**

Folder: GA-feature-subset-selection

File: GA algorithm for feature subselection

Description: The code implements a genetic algorithm approach for feature selection in machine learning using linear regression as the base model

Folder: Voting-Regressor

File: Feature importance ranking

Description: This code implements the calculation of feature importance of all features by using the dataset of interest fitted in Decision Tree model

File: Bayesian optimization

Description: The code will optimize each model separately using Bayesian Optimization and provide the best parameters and score for each model

File: Voting Regressor with 5 models

Description: This code utilizes a voting Regressor to ensemble five tree-based models, each with optimized hyperparameters, in order to achieve the best predictive capability for the model

Folder: Auto-data-splitting-by-article-ID

File: Auto data splitting by article ID

Description: Performs splitting of the database into test and training datasets from distinct sources (article ID)

File: Bayes_opt + CV by auto datasplitting by article ID

Description: The code employs Bayesian optimization to optimize the hyperparameters of the XGBoost model on diverse datasets obtained through the 'extract_rows' function of the 'Auto data splitting by article ID.py'

S1. Single-parameter linear regression analysis (features and super-features)

For the single-parameter linear regression analysis of (feature, output) couples, the dataset was shifted to (feature', output') couples to permit the use of a Log-Log scale (decimal log), and the linear regression was applied to this shifted Log-Log scale dataset. The sketch of the procedure for adaptation of (feature, output) or (super-feature, output) data to the Log-Log plot, so that the linear regression of the dataset can be performed, is described in Figure S1. A Log-Log plot is required for applying the linear regression also when there is no information on the occurrence of linear or nonlinear correlation in the dataset. However, some data have negative or 0 values (Figure S1A), which cannot be converted directly to a Log-Log scale. To avoid the exclusion of data ≤ 0 from the Log-Log plot, each list was rigidly shifted to ensure that its minimum become > 0 (Figure S1B). The procedure worked in two steps: (i) each list was shifted so that the minimum of each list become 0; (ii) each list was shifted again so that the minimum of each list become 1/20 of the penultimate value, leading to the final (feature', output') or (super-feature', output') datasets. Finally, these shifted but complete datasets were used for the linear regression (Figure S1C).

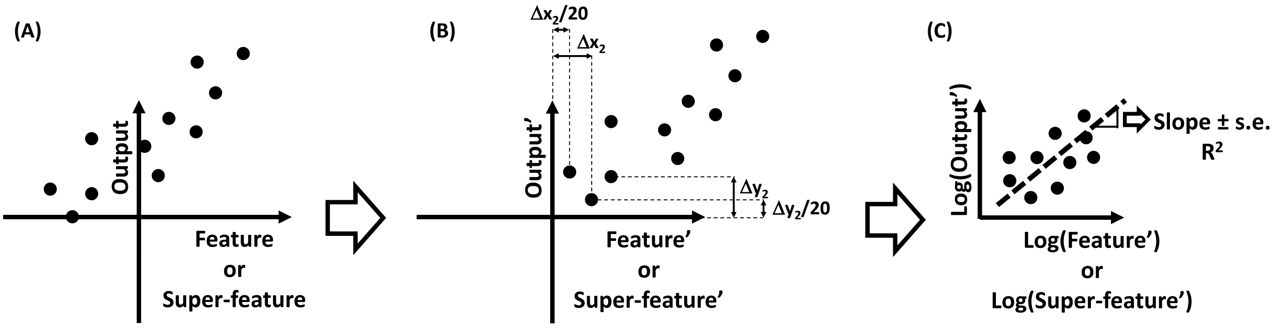

Figure S1. Sketch of the procedure for adaptation of (feature, output) or (super-feature, output) data to the Log-Log plot (decimal log) before the linear regression of the dataset. Some data have negative or 0 values (A), which cannot be converted directly to a Log-Log scale. To avoid the exclusion of data ≤ 0 from the Log-Log plot, each list was rigidly shifted to ensure that its minimum become > 0 (B). The procedure worked in two steps: (i) each list was shifted so that the minimum of each list become 0; (ii) each list was shifted again so that the minimum of each list become 1/20 of the penultimate value, leading to the final (feature', output') or (super-feature', output') datasets. Finally, these shifted but complete datasets were used for the linear regression (C).

From the linear regression of (feature', output') datasets on a Log-Log scale, easily understandable parameters such as the R^2 and the standard error (s.e.) on the slope of the linear fit were identified (Figure S2A-B). The R^2 and the relative s.e. of the slopes indicates that LAL features have low correlation with the oxidation state, hence the cross-correlations with the output are non-trivial. The features with the highest R^2 and the lowest relative s.e. are P12: % of O+Cl+CN+S of solvent (0.1647, 15 %), P11: # of atoms of solvent molecules (0.1489, 16 %), and P23: Density of solvent (0.1282, 17 %). The R^2 values are below the limit of statistical significance, which is indicative of the difficulty to identify single physical-chemical quantities responsible for LAL products output. In effect, the plot

for P12 (dashed red lines, Figure S2C) shows a dataset arranged in three sub-groups which cannot be described by a linear trend. If, on one hand, the largest R^2 is meaningful of the dominant role of the % of O+Cl+CN+S in solvent molecules for the oxidation state of Cu, on the other hand the grouping of the (P12', output') dataset suggests that cross-correlations with other features are indispensable to determine the output.

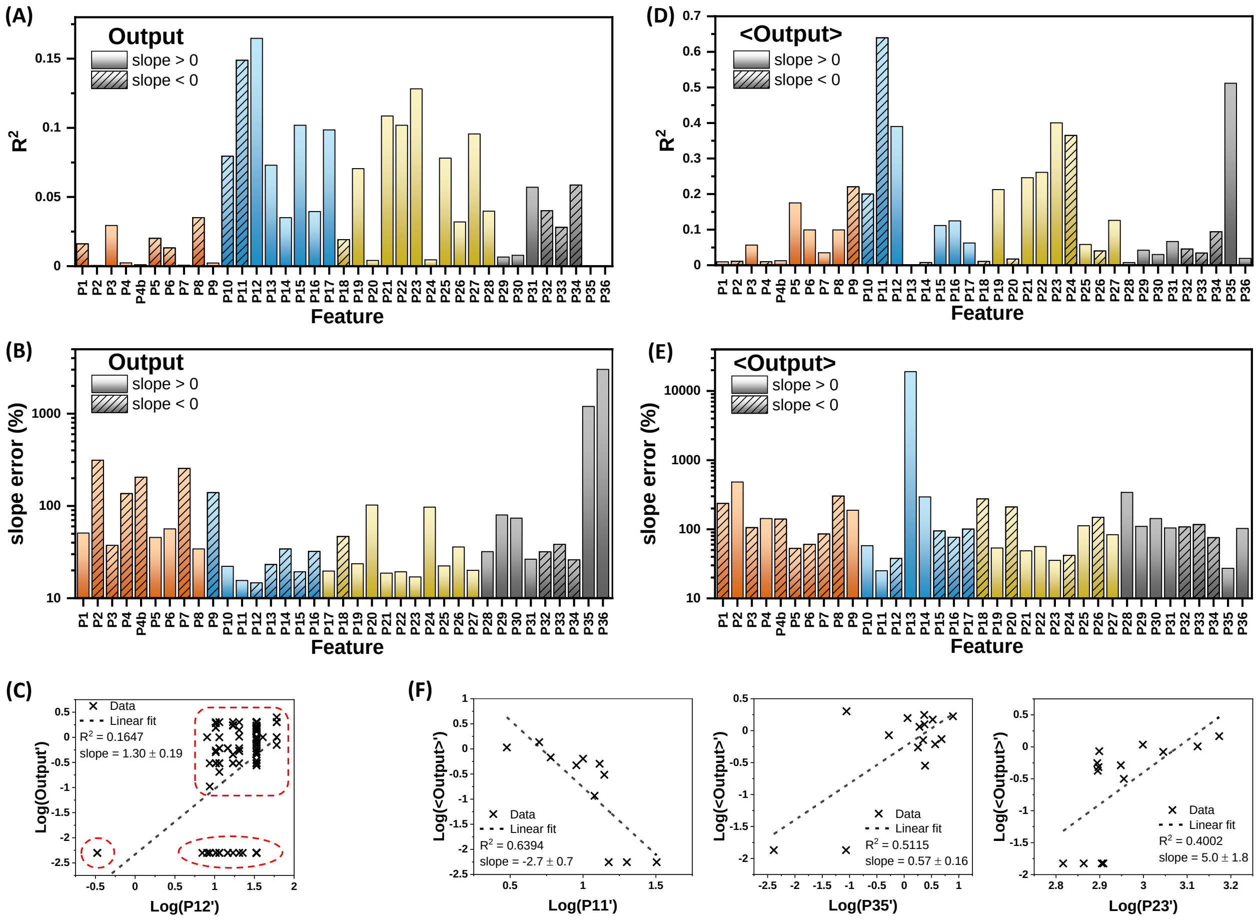

Figure S2. R^2 (A) and s.e. (%) on the slope (B) for the linear regression of single feature analysis. (C) Log-Log plot for the feature P12 with the best correlation with Cu oxidation state. The dashed lines identify three subsets of data which are not fitted simultaneously by the model. (D-E) R^2 (D) and s.e. (%) on the slope (E) for the linear regression of single feature analysis versus the average of the Cu oxidation state for each feature value (<output>). (F) Log-Log plot for the three features (P11, P35, P23) with the best correlation with the average Cu oxidation state for each feature value.

It should be noted also that in the literature some synthesis parameters are much more frequent than others. This leads to the inhomogeneous distribution of points in the features space with the accumulation of many points at the most frequent features, strongly affecting the results of the linear regression. Hence, the datasets of the average output (<output>) for each feature were also calculated and used for the linear regression. This average is obtained considering that, for each feature P_i in our database, there will be a number $N(P_i = \delta)$ of entries $P_{i,j}$ with the same value δ, and the corresponding $\langle output \rangle_\delta$ is obtained by dividing the sum of all the $\langle output \rangle_{\delta j}$ by $N(P_i = \delta)$

$$\langle output \rangle_{\delta} = \sum_{j}^{N(P_i=\delta)} \frac{output_{\delta j}}{N(P_i = \delta)} \qquad \text{(eq. S1)}$$

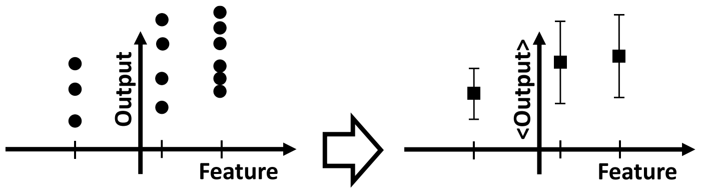

Figure S3. Sketch of the procedure for the calculation of (feature, <output>) couples, which are subsequently transformed in (feature’, <output>’) couples as described in Figure S1 and fitted with a linear regression with the results reported in Figure 2D-E-F.

Then, the linear regression was performed on the (Log[feature’], Log[<output>’]) couples obtained as described in Figure S1. In this way, the linear regression equally weighted all features, independent of the frequency of their use in the literature. The results show a net increment of the R^2 for several features (Figure S2D) but also an increment of the relative s.e. (Figure S2E) because of the lower number of data points in the linear fit. The features with the highest R^2 and the lowest relative s.e. are P11: # of atoms of solvent molecules (0.6394, 25 %), P35: Minimum ionization potential of the solute (0.5115, 27 %), P23: Density of solvent (0.4002, 35 %) and P12: % of O+Cl+CN+S of solvent (0.3898, 38 %). By crossing the results with those of Figure S2A-B, we confirmed that P11, P12 and P23 are the features with the strongest correlation with the oxidation state of the Cu NPs.

The regression analysis on single features does not tell if a combination of features is correlated with the output due to a synergic effect. For instance, literature analysis indicated that the oxidation state of Cu in NPs obtained in organic solvents may be lower when an inert gas atmosphere is used instead of ambient air, due to the presence of oxygen,[1] meaning that the combination of features of solvent and gas atmosphere is correlated with the average oxidation state.

Hence, the super-features SP_j were generated according to

$$SP_j = \prod_{i=1}^{N_p} P_i^{a_{ij}} \quad \text{(eq. S2)}$$

where N_P is the total number of single features P_i considered, a_{ij} is their exponent taken from the combination

$$A_j = (a_1, \ldots, a_i, \ldots, a_{Np}) \quad \text{(eq. S3)}$$

with j the index identifying the given combination among all those possible for the N_P features with one among three possible exponents (numerator: 1, denominator: -1, absent: 0). The number of combinations scales as 3^{Np} and is of the order of 1.5 10^{17} for $N_P = 36$. This was too much for the available computational capabilities. Hence, the features were initially divided into the four groups

G1-4: the laser source and other set-up parameters (G1 except P4b for insufficient data, N_P = 9), chemical properties of the liquid (G2, N_P = 8), physical properties of the liquid (G3, N_P = 11), properties of the prevailing solute (G4, N_P = 8). These groups were screened for the SP_j with the best correlation under a linear regression, leading to the identification of other subsets of features labelled SP_C (all features with exponents different from 0 in the super-features with the highest R^2 obtained from SP_G1, SP_G2, SP_G3 and SP_G4) and SP_D (all the features with average exponent different from 0 in the combinations with the 100 highest R^2 obtained from SP_G1, SP_G2, SP_G3 and SP_G4). Moreover, also the sixteen features with the highest R^2 in the histogram of Figure S2A and Figure S2D were considered, labelled respectively SP_A and SP_B. Other combinations (SP_E, SP_F, SP_G, SP_H, SP_I) were also assessed taking the values with exponents systematically different from 0 in the super-feature with the highest R^2 obtained from SP_A, SP_B, SP_C and SP_D. The results (see Figure S4 and Tables S1-2 in S.I.) indicate an increment of R^2 when passing from single features (P_i) to their combinations SP_j. SP_E, SP_F, SP_H and SP_I all have an R^2 slightly larger than 0.3 and s.e. < 10 %. We hypothesize that errors in the literature data, due to the experimental variables that cannot be accounted for in the database such as NPs ageing, target ageing and inaccurate assessment of Cu NPs composition, surely contribute to the low R^2, by lowering the possible correlation between the output and the super-features.

According to the linear regression analysis with the super-features, the best result (R^2 = 0.3122, s.e. = 9.6 %) is achieved for

$$SP\_F = \frac{P9P17P21P23P33}{P8P10P11P14P19P22P26P35} \qquad \text{(eq. S4)}$$

and the best equation obtained by this procedure for the prediction of Cu oxidation state in NPs from LAL is

$$Log(Output') = \text{-}0.40 \pm 0.05 + (0.99 \pm 0.10) Log(SP\_F') \qquad \text{(eq. S5).}$$

However, the R^2 is low and the dataset for SP_F is still divided into three groups as observed for P12 (dashed red lines in Figure S4C). This suggests the absence of a physical or chemical reason for expecting a simple linear correlation between the Log of features or super-features and the oxidation states of Cu NPs. Instead, Figure S4C indicates that the relationship is much more complex and the cross-correlations between features and Cu oxidation state are not completely accessible with the linear regression approach.

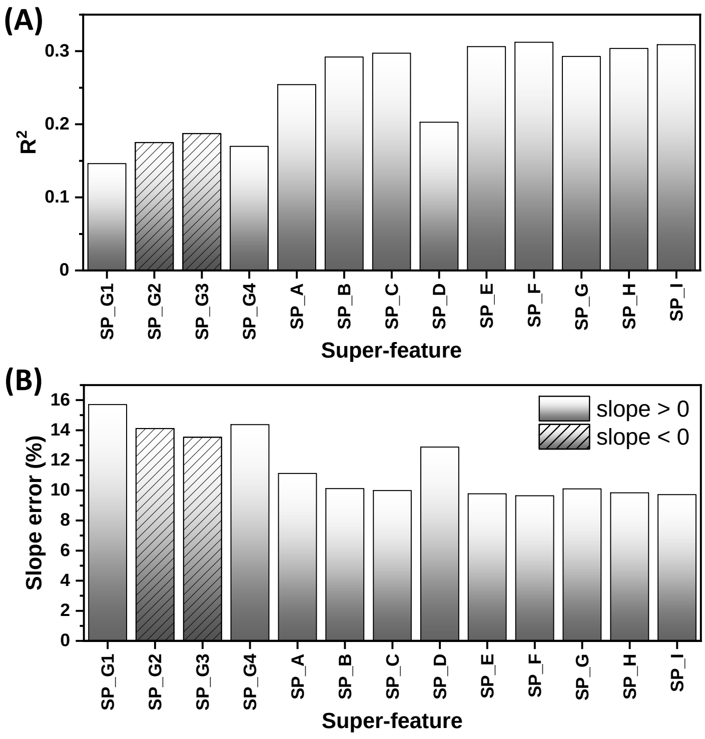

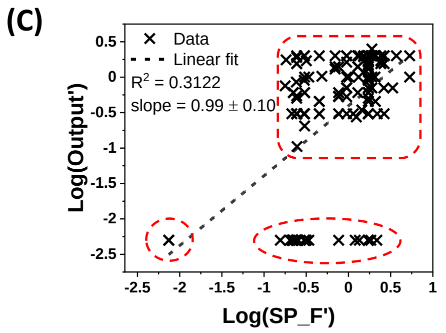

$$Log(Output') = -0.40 \pm 0.05 + (0.99 \pm 0.10) Log(SP\_F')$$

$$SP\_F = \frac{P9P17P21P23P33}{P8P10P11P14P19P22P26P35}$$

Figure S4. R^2 (A) and s.e. (%) on the slope (B) for the linear regression of the super-features analysis. (C) Log-Log plot for the super-feature SP_F, also shown in the figure, which has the best correlation with the oxidation state of Cu, as described by the equation below the plot. The dashed lines identifiy three subsets of data which are not fitted simultaneously by the model.

Table S1. Summary of super-features (SP_j) used for the linear regression analysis. For each feature included in the SP_j the exponent (a_{ij}) which maximised the R^2 of the linear regression of the (SPj', output') datasets are reported.

	SP_G1	SP_G2	SP_G3	SP_G4	SP_A	SP_B	SP_C	SP_D	SP_E	SP_F	SP_G	SP_H	SP_I
**G1: Set-up parameters**													
P1. Pulse duration (s)	0							0			0		
P2. Wavelength (nm)	0										-1	0	
P3. Repetition rate (Hz)	0							1					
P4. Pulse energy (J/pulse)	0							0					
P5. Lens focal length (cm)	0					0		1					
P6. Duration of synthesis (min)	0				1	1		1	0				
P7. # pulses	0				0			1			1	0	0
P8. Type of cell	0				-1	-1		-1		-1	-1	-1	-1
P9. Gas electron affinity (eV)	-1					1	1	1	1	1	-1	1	1
**G2: Chemical properties of liquid**													
P10. MW (Da)		0			-1	-1				-1			
P11. # of atoms		1				-1	-1		-1	-1	-1	-1	-1
P12. % of O+Cl+CN+S		0				0					0		
P13. Minimum bond energy (kJ/mol)		0											
P14. Average bond energy (kJ/mol)		1					-1		-1	-1		-1	-1
P15. Maximum electron affinity (eV)		0				1			0		1		
P16. Minimum ionization potential (eV)		0			-1	1			1	0		1	1
P17. Ionization potential (eV)		1					1		1	1		0	0
**G3: Physical properties of liquid**													
P18. Refractive index at 589 nm			1				1			0			
P19. Relative dielectric constant			0		1	-1			-1	-1		-1	-1
P20. Viscosity (mPa s)			0								0		
P21. Henry constant (MPa)			0			1			1	1	1	1	1
P22. Surface tension (mN/m)			0		0	-1			-1	-1	1	-1	-1
P23. Density (kg/m3)			-1		1	1	1		1	1	1	1	1
P24. Boiling point (K)			0		1	0							
P25. Melting point (K)			1		0		0						
P26. Specific heat capacity (J/mol K)			1		-1		-1		-1	-1		-1	-1
P27. Thermal conductivity (W/K m)			-1		-1	-1	1		1	0	1	1	1
P28. Sound speed (m/s)			0										
**G4: Properties of prevailing solute**													
P29. MW				-1			0	0					
P30. # of atoms				0	0			1					
P31. % of O+Cl+CN+S				0	0			1			1		0
P32. Minimum bond energy (kJ/mol)				1	0		0	-1					
P33. Average bond energy (kJ/mol)				1	-1		1	-1	1	1		1	1
P34. Maximum electron affinity (eV)				1		1	0	1	0		0		

P35. Minimum ionization potential (eV)				1			-1	-1	-1	-1	0	-1	-1
P36. Mass fraction in solution				-1			0	-1					

Table S2. Summary of linear regression analysis for the various super-features tested.

	SP_G1	SP_G2	SP_G3	SP_G4	SP_A	SP_B	SP_C	SP_D	SP_E	SP_F	SP_G	SP_H	SP_I
**R^2**	0.14614	0.17482	0.18728	0.16966	0.25422	0.29196	0.29719	0.20277	0.30626	0.31224	0.29272	0.3037	0.30886
**slope**	1.31	-2.4	-3.1	1.21	0.67	0.46	1.16	0.08	1.13	0.99	0.24	0.98	0.97
**s.e.**	0.21	0.3	0.4	0.17	0.07	0.05	0.12	0.01	0.11	0.10	0.02	0.10	0.09
**s.e. (%)**	15.7	14.1	13.5	14.4	11.1	10.1	10.0	12.9	9.8	9.6	10.1	9.8	9.7

S2. Comparison of the ML performances with the top 9, 10 and 11 features

The ML models were optimised also by including the 10th and 11th feature in the ranking of Table S4, which belong to G3 about the physical properties of the solvent (P18: Refractive index at 589 nm, P24: Boiling point). The results, summarized in Figure S5, indicate stable or worst performance when passing from 9 to 10 features and 11 features, in agreement with the "curse of dimensionality" of ML models.[2–6] Therefore, the increase of the number of feature beyond 9 is unnecessary and not insightful about the synthesis process.

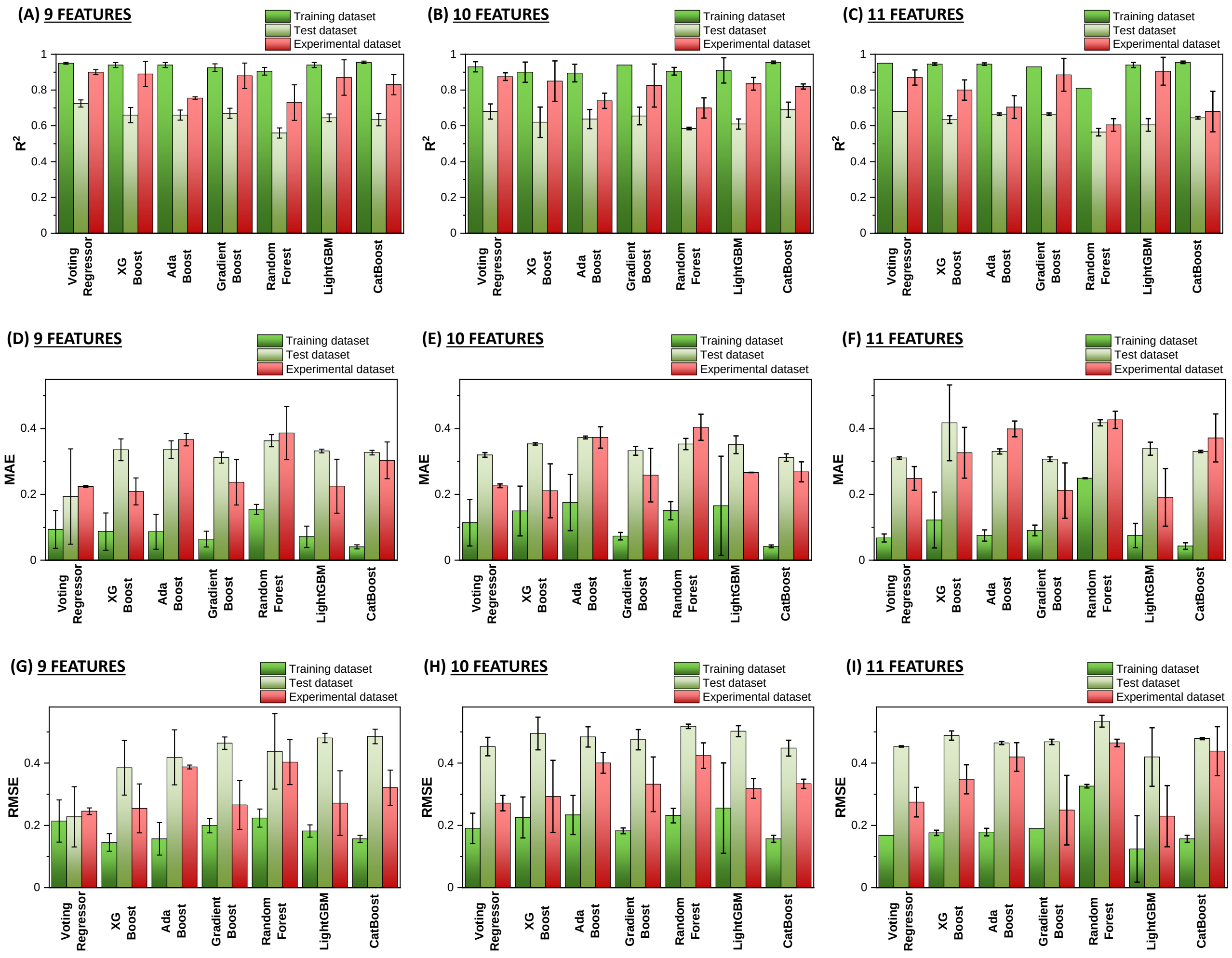

Figure S5. (A-C) R^2 of the linear fit of the predicted values versus real values for the training and test datasets taken from the literature and using the best 9, 10 or 11 features resulting from the ranking in Table S4. Red bars report the R^2 of the linear fit for the predicted values versus the values obtained from the experiments in this study. MAE (D-F) and RMSE (G-I) are also reported showing that the models maintain more stable or better performance with 9 features.

S3. Validation of the ML model with dataset splitting for different sources

The Figure S6 below reports the average on the best five results achieved with the ML models after operating on the training and test datasets to avoid that the same article (literature source) contributed simultaneously to the two groups of input. For the sake of comparison, the same features, models for the Voting Regressor and hyperparameters resulting from the analysis with the unsplitted database were used. Despite the splitting, the performance of the Voting Regressor model is similar to that without the splitting. The general decrease of the coefficient of determinations is justified by the small size of the database and by the statistical advantage introduced in such a critical case by the use the same literature sources for both the training and input datasets, because of the intra-laboratory homogeneity in the experimental synthetic procedures and in the methodologies for the assessment of products.

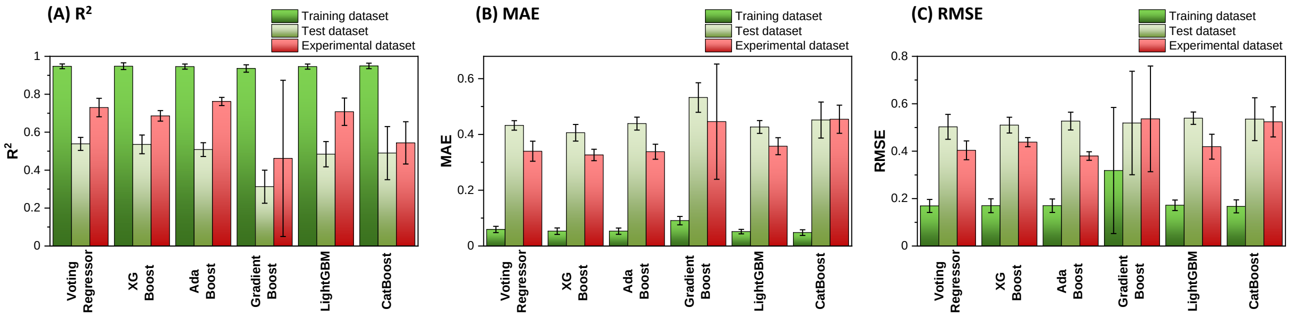

Figure S6. Average R^2 (A), MAE (B) and RMSE (C) on the best five training and test inputs obtained after splitting for different sources. Red bars report the R^2 of the linear fit, MAE and RMSE for the predicted values versus the values obtained from the experiments in this study.

S4. Prediction of Cu oxidation state in high throughput LAL conditions

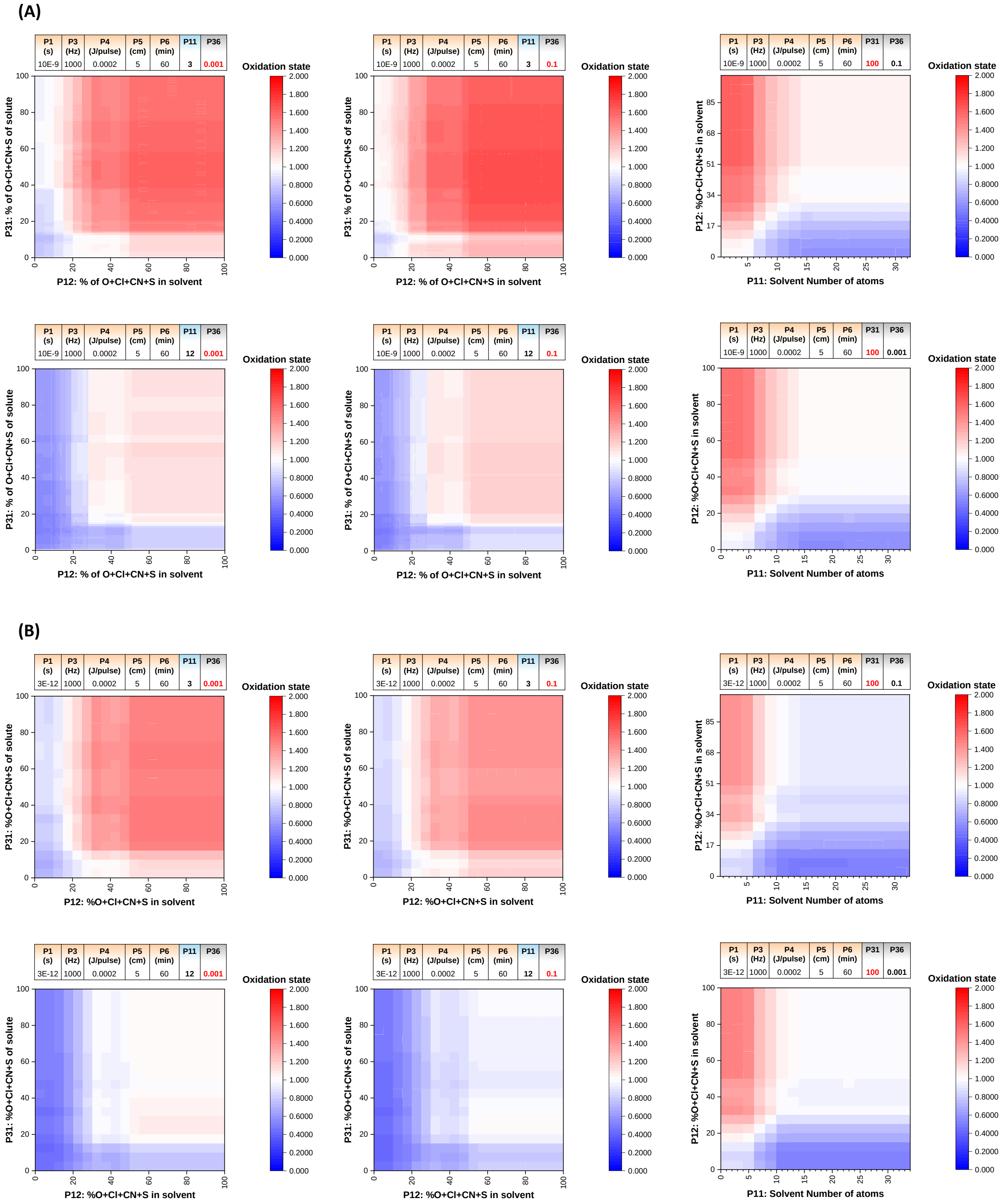

(C)

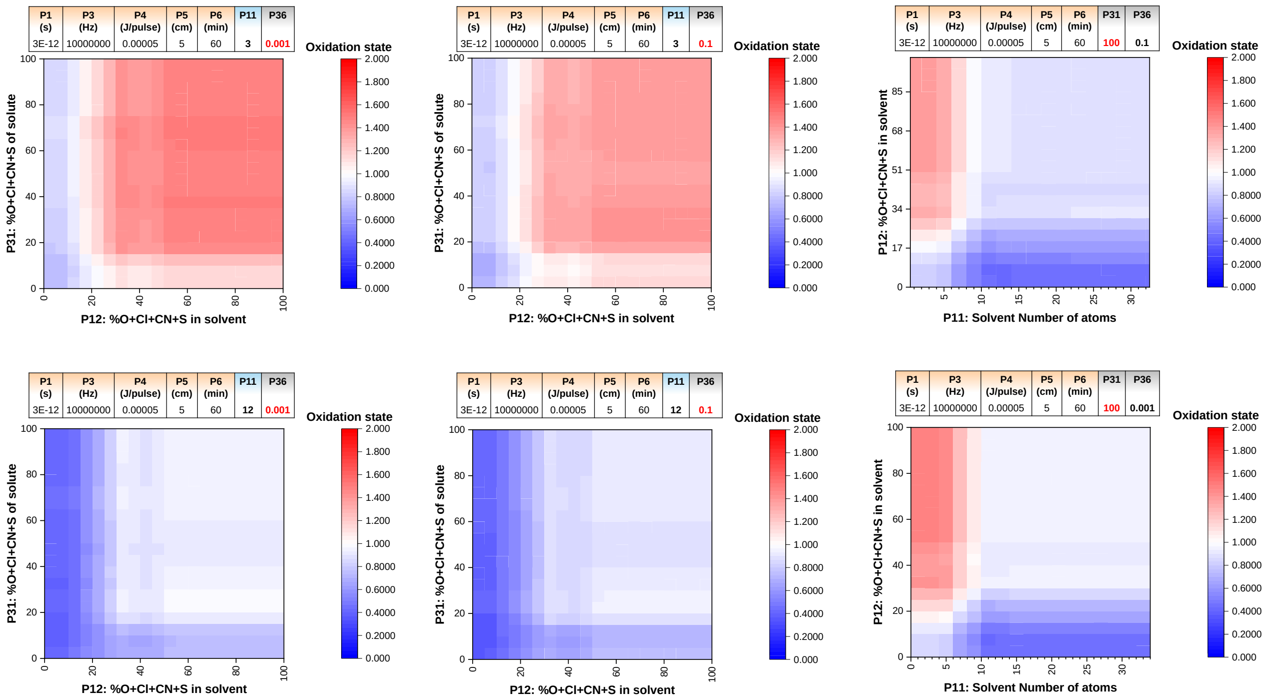

Figure S7. Prediction of the variation of the oxidation state as a function of the % of O+Cl+CN+S in solvent molecules (P12) and solute molecules (P31) at two solute concentrations (P36 = 0.001 and 0.1) and two different numbers of atoms in solvent molecules (P11: 3 and 12), and as a function of the number of atoms (P11) and of the % of O+Cl+CN+S (P12) in solvent molecules for a % of O+Cl+CN+S in solute (P31) of 100 and two solute concentrations (P36 = 0.1 and 0.001). Set-up parameters are fixed as reported in the tables above each graph considering typical experimental conditions for kHz LAL with ns (A) or ps (B) pulses and MHz LAL (C). All predictions are obtained with the best model (Voting Regressor).

The general trend of the predictions is similar to the results in Figure 6 of the main article, where ns laser pulses with repetition rate of 50 Hz and energy in the range of mJ/pulse are adopted. However, the predictions for a higher repetition rate and lower pulse energy indicate a slight increase of the oxidation state, which can be reduced by shortening the pulse duration from ns to ps.

References

(1) Marzun, G.; Bönnemann, H.; Lehmann, C.; Spliethoff, B.; Weidenthaler, C.; Barcikowski, S. Role of Dissolved and Molecular Oxygen on Cu and PtCu Alloy Particle Structure during Laser Ablation Synthesis in Liquids. *ChemPhysChem* **2017**, *18* (9), 1175–1184.

(2) Wang, Z.; Sun, Z.; Yin, H.; Liu, X.; Wang, J.; Zhao, H.; Pang, C. H.; Wu, T.; Li, S.; Yin, Z.; Yu, X. F. Data-Driven Materials Innovation and Applications. *Adv. Mater.* **2022**, *34* (36), 2104113.

(3) Gao, C.; Min, X.; Fang, M.; Tao, T.; Zheng, X.; Liu, Y.; Wu, X.; Huang, Z. Innovative Materials Science via Machine Learning. *Adv. Funct. Mater.* **2022**, *32* (1).

(4) Bischl, B.; Binder, M.; Lang, M.; Pielok, T.; Richter, J.; Coors, S.; Thomas, J.; Ullmann, T.; Becker, M.; Boulesteix, A. L.; Deng, D.; Lindauer, M. Hyperparameter Optimization: Foundations, Algorithms, Best Practices, and Open Challenges. *Wiley Interdiscip. Rev. Data Min. Knowl. Discov.* **2023**, *13* (2), e1484.

(5) Berisha, V.; Krantsevich, C.; Hahn, P. R.; Hahn, S.; Dasarathy, G.; Turaga, P.; Liss, J. Digital Medicine and the Curse of Dimensionality. *npj Digit. Med. 2021 41* **2021**, *4* (1), 1–8.

(6) Chen, L. Curse of Dimensionality. *Encycl. Database Syst.* **2009**, 545–546.
